# Evolutionary diversification of acyl-CoA synthetases underpins hydrophobic barrier formation across diverse tomato tissues and beyond

**DOI:** 10.1093/hr/uhaf114

**Published:** 2025-04-28

**Authors:** Jianfeng Jin, Qiyu He, Xiangyi Feng, Jianjing Wang, Tao Lyu, Jinheng Pan, Jiarong Chen, Shan Feng, Xing-xing Shen, Jingquan Yu, Robert L Last, Pengxiang Fan

**Affiliations:** Department of Horticulture, Zijingang Campus, Zhejiang University, Hangzhou 310058, China; Department of Horticulture, Zijingang Campus, Zhejiang University, Hangzhou 310058, China; Department of Horticulture, Zijingang Campus, Zhejiang University, Hangzhou 310058, China; Department of Horticulture, Zijingang Campus, Zhejiang University, Hangzhou 310058, China; Department of Horticulture, Zijingang Campus, Zhejiang University, Hangzhou 310058, China; Mass Spectrometry & Metabolomics Core Facility, The Biomedical Research Core Facility, Westlake University, Hangzhou 310030, China; Key Laboratory of Biology of Crop Pathogens and Insects of Zhejiang Province, Institute of Insect Sciences, Zhejiang University, Hangzhou 310058, China; Mass Spectrometry & Metabolomics Core Facility, The Biomedical Research Core Facility, Westlake University, Hangzhou 310030, China; Key Laboratory of Biology of Crop Pathogens and Insects of Zhejiang Province, Institute of Insect Sciences, Zhejiang University, Hangzhou 310058, China; Department of Horticulture, Zijingang Campus, Zhejiang University, Hangzhou 310058, China; Department of Biochemistry and Molecular Biology, Michigan State University, East Lansing, MI 48824, USA; Department of Plant Biology, Michigan State University, East Lansing, MI 48824, USA; Department of Horticulture, Zijingang Campus, Zhejiang University, Hangzhou 310058, China; Key Laboratory of Horticultural Plants Growth and Development, Agricultural Ministry of China, Hangzhou 310058, China

## Abstract

The transition of plants from aquatic to terrestrial environments required effective barriers against water loss and UV damage. The plant cuticle, a hydrophobic barrier covering aerial surfaces, emerged as a critical innovation, yet how its biosynthesis is regulated in specialized structures remains poorly understood. This study identifies two long-chain acyl-CoA synthetases, SlLACS1 and SlLACS2, that exhibit both distinct and overlapping functions in cuticle formation across tomato tissues. These genes show striking specificity in different trichome types: *SlLACS1* functions in type I/IV trichomes, while *SlLACS2* is required for type VI trichome cuticle integrity. However, they act redundantly in leaf epidermal and fruit cuticle formation, as revealed by analysis of single and double mutants. Unexpectedly, simultaneous disruption of both genes severely compromises pollen viability through defective pollen coat formation. Biochemical characterization demonstrates that SlLACS1 and SlLACS2 maintain their ancestral enzymatic function of activating long-chain fatty acids, an activity conserved from algal LACS homologs. These findings reveal how gene duplication and diversification facilitated the development of specialized hydrophobic barrier functions in distinct tissues while maintaining redundancy in fundamental protective structures, representing a sophisticated adaptation to terrestrial life.

## Introduction

The evolutionary transition from aquatic algal ancestors to terrestrial land plants, which occurred approximately 450 million years ago, fundamentally transformed life on Earth [1–3]. This adaptation shift in habitat exposed early land plants to a myriad of environmental challenges, most notably desiccation and increased exposure to ultraviolet (UV) radiation [4, 5]. As an adaptive strategy, early land plants developed a diverse array of protective mechanisms, notably the development of hydrophobic layers in their epidermal cells, such as the cuticle [6, 7]. These layers were critical in protecting from water loss and shielding against UV radiation, thus facilitating the adaptation of these plants to terrestrial environments [8].

As terrestrial plants evolved, they underwent a substantial increase in tissue and organ complexity, evolving from the simple, undifferentiated thalli of their ancestors to the highly specialized and diverse structures observed in modern angiosperms [3, 9–11]. The advent of varied leaf structures and highly specialized reproductive organs, such as flowers and seeds, marked significant innovations to facilitate the plant reproductive strategies [9, 12–14]. Concurrently, the hydrophobic layers of the epidermal cells evolved to coat these increasingly complex tissues, ensuring enhanced protection against water loss and UV radiation.

Despite the recognized importance of hydrophobic layers in plant adaptation to terrestrial environments, pertinent questions remain. Firstly, it is uncertain whether these layers uniformly coat all tissue types. One such example is that the presence of cuticular layers on trichomes—the protruding epidermal cells forming hair-like structures on plant surfaces—has not been thoroughly examined. Trichomes, part of the plant defensive epidermal layer, are broadly categorized into non-glandular and glandular types [15–17]. Glandular trichomes are particularly intriguing for their secretion of specialized metabolites that deter insect herbivores [18, 19]. In tomato (*Solanum lycopersicum*), glandular trichomes are categorized into multicellular type VI trichomes, producers of volatile terpenoids, and type I/IV trichomes, producers of defensive acylsugars [20, 21]. These trichomes serve as a first line of protection against herbivory. However, it remains unclear whether a hydrophobic layer is essential for their effective functioning.

Second, the evolutionary mechanisms underlying the biosynthesis of epidermal hydrophobic layers from fatty acid metabolism remain elusive. Current insights into hydrophobic layers come mainly from studies on the cuticles of Arabidopsis leaf epidermis and tomato fruit [22–24]. The plant cuticle, a hydrophobic layer coating the epidermis of plant parts, is composed of cutin and cuticular wax [25, 26]. Originating from fatty acid metabolism, both components are made up of fatty acid chains: cutin is primarily a polymer of ω- and mid-chain hydroxy and epoxy C16 and C18 fatty acids, while cuticular wax is a complex mixture of straight-chain C20 to C60 aliphatics [27]. Cuticle component biosynthesis starts with the activation of fatty acids by long-chain acyl-CoA synthetase (LACS) enzymes, which convert free fatty acids to fatty acyl-CoAs [28]. These activated intermediates are then elongated, hydroxylated, and further modified by a series of enzymes, including fatty acid elongases (FAEs), cytochromes P450 (CYPs), and glycerol-3-phosphate acyltransferases (GPATs), to form the cutin and wax building blocks [26, 27]. LACS catalyze a critical early step in cuticle biosynthesis by activating fatty acids for subsequent modifications. In Arabidopsis, two members of this nine-gene family, *AtLACS1* and *AtLACS2*, specifically contribute to cuticle development in leaves and flowers [28]. Although LACS function is well characterized in Arabidopsis, understanding how these enzymes evolved to regulate hydrophobic barrier biosynthesis across diverse tissue types and species, including economically important crops like tomato, remains a fundamental challenge.

In this study, transcriptome analysis of dissected tomato leaf epidermis identified two key genes, *SlLACS1* and *SlLACS2*, which exhibit both specialized and overlapping functions in cuticle biosynthesis. While these genes show striking specificity in different trichome types—*SlLACS1* in type I/IV and *SlLACS2* in type VI trichomes—they function redundantly in maintaining cuticle integrity in leaves and fruits. Genetic analysis revealed that disruption of both genes severely compromises not only vegetative barriers but also pollen viability, highlighting their essential role in reproductive success. These findings demonstrate how two LACS genes with conserved biochemical activities have evolved distinct tissue-specific functions while maintaining overlapping roles in fundamental protective barriers, illustrating a sophisticated adaptation to terrestrial life.

## Results

### Identification of key genes involved in cuticle biosynthesis of tomato leaf epidermis

**Figure 1:**
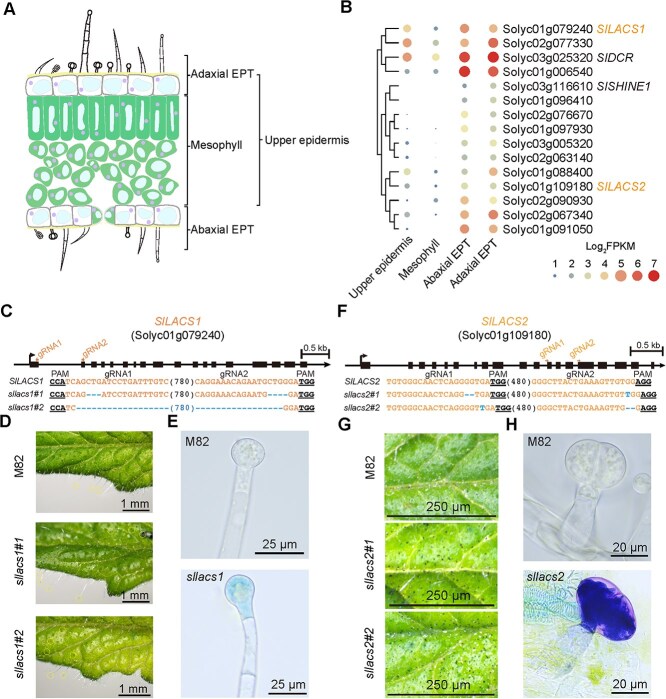
Increased permeability in type I/IV trichomes of *SlLACS1* mutants and in type VI trichomes of *SlLACS2* mutants evidenced by TB staining. A) Diagram of the four leaf tissues dissected for comparative transcriptomic analysis, labeled as adaxial EPT (adaxial epidermis with trichomes), abaxial EPT (abaxial epidermis with trichomes), mesophyll, and upper epidermis. B) The previous characterized tomato fruit epidermal cuticle biosynthesis-related genes, *SlDCR* (Solyc03g025320) and *SlSHINE1* (Solyc03g116610), and the putative tomato trichome cuticle biosynthesis-related genes, *SlLACS1* (Solyc01g079240) and *SlLACS2* (Solyc01g109180), cluster together with 15 co-regulated genes preferentially expressed in adaxial EPT, as shown in the heatmap. The heatmap shows the expression value of the 15 genes in different tissues. C) Schematic representation of gRNA target sites on *SlLACS1* (arrows and orange text), with PAM sites underlined. Resulting *SlLACS1* mutations in *sllacs1#1* and *sllacs1#2* mutants are denoted in dotted line. D) TB staining of leaf trichomes indicates that type I/IV trichomes were stained in *sllacs1* mutants, contrasting with the unstained M82 control. Stained trichomes are circled. Scale bars = 1 mm. E) Magnified images of type I/IV glandular trichomes from M82 and *sllacs1* plants post TB staining show TB uptake in the trichomes of *sllacs1* mutants. Scale bars = 25 μm. F) CRISPR-Cas9 strategy for *SlLACS2* disruption. Schematic representation of gRNA target sites on *SlLACS2* (arrows and orange text), with protospacer adjacent motif (PAM) sites underlined. Resulting *SlLACS2* mutations in *sllacs2#1* and *sllacs2#2* mutants are denoted in dotted line. G) TB staining of leaves shows the presence of tiny blue dots identified as the type VI trichomes on the surface of *sllacs2* mutants, which are not observed in the M82 control. Scale bars = 250 μm. H) Enlarged images of type VI glandular trichomes from M82 and *sllacs2* plants post TB staining reveal TB uptake in the trichomes of *sllacs2* mutants. Scale bars = 20 μm.

The cuticle, forming the outermost hydrophobic barrier of plant epidermis, consists of fatty acid-derived compounds, including cuticular waxes and cutin [29]. In tomatoes, the majority of cuticle biosynthesis research has focused on fruit epidermis [22, 23, 30–33], with limited studies addressing tomato leaves, which host a diverse array of glandular trichomes, markedly different from Arabidopsis leaf types. We hypothesized that expression of genes crucial for leaf cuticle biosynthesis would be enriched in the epidermal outer layer. To identify these candidate genes, we divided the tomato leaves into four layers through microscopic dissection and conducted weighted correlation network analysis (WGCNA) on their differentially expressed genes. The dissected layers included adaxial epidermis with trichomes (adaxial EPT), abaxial epidermis with trichomes (abaxial EPT), mesophyll, and the upper epidermis with a combination of adaxial EPT and mesophyll (Fig. 1A). A dedicated website was developed to host the Tomato Leaf Transcriptome Datasets (TLTD) for community access and exploration of gene expression patterns in tomato epidermis (https://sol-slod.net/).

The WGCNA revealed a 'blue module' comprising 1061 highly correlated genes that were enriched in adaxial EPT tissue (Fig. S1A; Table S1). Further refinement based on stringent criteria (FPKM ≥10 and log_2_[adaxial EPT/mesophyll] ≥ 2) narrowed these candidates to 50 genes encoding transcription factors from ERF, MYB, and HD-ZIP families, as well as catalytic enzymes from hydrolase, ligase, lyase, oxidoreductase, and transferase protein families (Fig. S1B and C; Table S2). Among them, the BAHD family acyltransferase *SlDCR* (DEFECTIVE IN CUTICULAR RIDGES, Solyc03g025320) and the AP2 domain transcription factor *SlSHN1* (SHINE1, Solyc03g116610) were previously implicated in tomato fruit epidermis suberin and wax biosynthesis [34, 35]. Therefore, we reasoned that other genes within this co-expression module, particularly those closely associated with *SlDCR* and *SlSHN1* (Fig. 1B; Table S3), might play roles in synthesizing the hydrophobic layer on tomato leaves.

**Figure 2:**
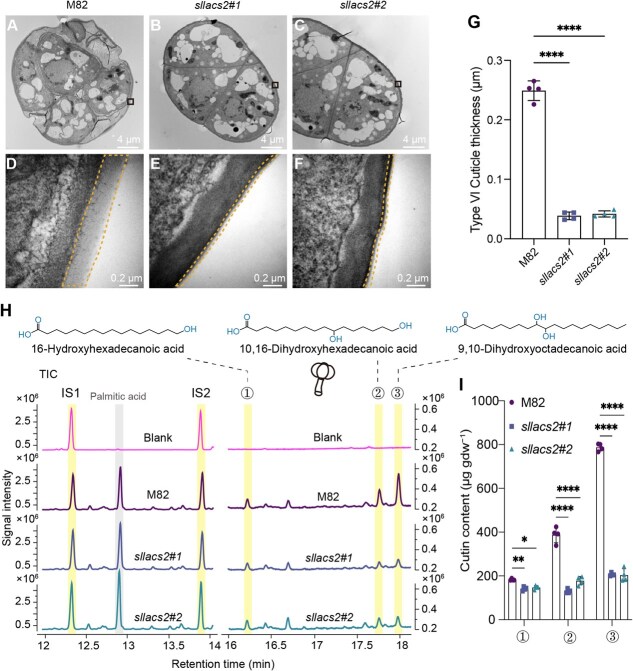
Altered cuticle ultrastructure and reduced cutin content in type VI trichomes of *sllacs2* mutant. A-C) Analysis of the type VI trichomes via TEM. The cross section of the glandular head of the type VI trichomes from M82 (A), *sllacs2#1* (B) and *sllacs2#2* (C) plants were examined. Scale bars = 4 μm. D-F) The enlarged TEM images showed that the cuticle layer was present in the tip cell of type VI trichomes from M82 (D), while this layer was significantly reduced in the type VI trichomes from the mutant *sllacs2#1* (E) and *sllacs2#2* (F), as indicated by the dotted lines. Scale bars = 0.2 μm. G) The cuticle layer thickness of type VI trichomes from different plants were measured and quantified. Data are means ± SD (*n* = 4); Unpaired *t*-test was performed, and significant differences are represented by black asterisks: **** *P* < 0.0001. H) Aligned total ion chromatogram (TIC) for cutin monomers (16-Hydroxyhexadecanoic acid, 10,16-Dihydroxyhexadecanoic acid, and 9,10-Dihydroxyoctadecanoic acid) detection by GC–MS analysis of type VI trichomes from blank, M82, *sllacs2#1* and *sllacs2#2*. IS, internal standard (IS1: ω-pentadecalactone, IS2: Methyl heptadecanoate). I) Quantification of cutin monomers content in the type VI trichome of M82, *sllacs2#1*, and *sllacs2#2* plants. Data are presented as means ± SD (*n* = 4); Unpaired *t*-test was performed and the significant differences are represented by black asterisks: ^*^  *P* < 0.05, ^**^  *P* < 0.01, ^****^  *P* < 0.0001.

To experimentally screen the genes involved in tomato leaf epidermal cuticle biosynthesis, we employed virus-induced gene silencing (VIGS) to suppress 12 co-expressed genes transiently. The integrity of the plant epidermis was then assessed using the toluidine blue (TB) staining method [36]. Among these silenced genes, *SlLACS1* (Solyc01g079240) and *SlLACS2* (Solyc01g109180)-silenced plants exhibited distinctive blue dots on their leaf surface following TB staining (Fig. S2), differing from the anticipated leaf epidermis defects. Closer examination showed that silencing *SlLACS1* and *SlLACS2* increased TB permeability specifically in glandular trichomes, with no apparent change in the leaf epidermal permeability (Fig. S2A). Our VIGS-based screening revealed an unexpected role of *SlLACS1* and *SlLACS2* in glandular trichome permeability, prompting further investigation using stable genetic approaches.

### Disruption of *SlLACS1* and *SlLACS2* leads to increased permeability in glandular trichomes due to impaired cutin biosynthesis

To validate the VIGS screening result*,* we generated stably inherited mutant targeting *SlLACS1* and *SlLACS2* in the tomato cultivar M82 using CRISPR/Cas9 technology. Two homozygous *sllacs1* mutants were obtained: *sllacs1#1* with a 3-bp and 4-bp deletion, and *sllacs1#2* with an 815-bp deletion, both predicted to cause frameshift and premature translation termination (Fig. 1C). Similarly, the homozygous mutant lines *sllacs2#1* and *sllacs2#2* exhibited differential patterns of deletions and insertions within the exon regions, leading to frameshift mutations (Fig. 1F). These genetic alterations are predicted to introduce premature stop codons in the coding sequence of *SlLACS2* (Fig. 1F). Subsequent TB staining analysis of the *sllacs1* and *sllacs2* mutants corroborated our VIGS results, showing no alterations in leaf epidermal permeability. However, the mutants exhibited an enhanced glandular trichome phenotype compared to VIGS-silenced plants, characterized by abundant dark blue dots across the leaf surface (Fig. 1D and G). Microscopic examination identified these TB-stained structures as type I/IV glandular trichomes in *sllacs1* mutants (Fig. 1E) and type VI glandular trichomes in *sllacs2* mutants (Fig. 1H). These results demonstrate that *SlLACS1* and *SlLACS2* disruption specifically increases TB permeability in the tip cell of type I/IV and type VI trichomes, respectively, while maintaining normal leaf epidermal barrier function.

To investigate the underlying mechanism of enhanced trichome permeability, we performed ultrastructural analysis using transmission electron microscopy (TEM). Given the complex architecture and technical challenges associated with preserving different trichome types during sample preparation, our initial attempts to analyze type I/IV trichomes were hindered by their fragile nature, as their unicellular tip cells could not maintain structural integrity during the dehydration steps required for TEM sample preparation. We therefore focused our detailed structural analysis on type VI trichomes, which possess more robust multicellular tip cells. TEM analysis of the type VI trichomes from *sllacs2* mutants revealed a significantly thinner cuticle-like structure compared to wild-type M82 (Fig. 2A–G), suggesting impaired cutin biosynthesis. While cuticle structures on trichomes have not been previously documented, we hypothesized that *SlLACS2* disruption might affect cutin accumulation in glandular trichomes. To further investigate this hypothesis, we took advantage of the easily isolatable nature of type VI trichomes and performed gas chromatography–mass spectrometry (GC–MS) analysis of cutin monomer composition using isolated type VI trichomes from both M82 and *sllacs2* mutants. The analysis revealed significant reductions in specific cutin monomers in *sllacs2* mutants, particularly 16-hydroxyhexadecanoic acid, 10,16-dihydroxyhexadecanoic acid, and 9,10-dihydroxyoctadecanoic acid (Figs 2H, I, and S3). These findings provide the first direct evidence for the presence of a cuticle layer on tomato type VI glandular trichomes and establish SlLACS2 as an essential component of trichome cutin biosynthesis. Given the similar TB staining phenotypes observed in *sllacs1* mutants (Fig. 1D and E) and the close homology between *SlLACS1* and *SlLACS2*, we hypothesize that SlLACS1 likely plays a parallel role in cutin production in type I/IV trichomes.

Given the importance of cultivated tomato type I/IV and VI glandular trichomes in plant defense through specialized metabolite production [37], we investigated whether the compromised trichome cuticle affected specialized metabolites accumulation. Considering that type I/IV trichomes are the primary sites for acylsugar production, a defensive metabolite [38], we first examined whether the *SlLACS1* disruption affects acylsugar accumulation. Analysis of *sllacs1* mutant leaves revealed acylsugar levels comparable to M82 controls (Fig. S4). We next investigated the impact of *SlLACS2* disruption on specialized metabolite profiles in type VI trichomes. GC–MS analysis of leaf terpenoid profiles revealed significantly reduced levels of both monoterpenes and sesquiterpenes in *sllacs2* mutants compared to M82 controls (Fig. S5A and B). Specifically, three monoterpenes (α-terpinene, D-limonene, and β-phellandrene; Fig. S5A) and three sesquiterpenes (δ-elemene, β-caryophyllene, and α-humulene; Fig. S5B) showed marked reductions. The compromised trichome cuticle likely facilitates the escape of these volatile compounds through the gas phase, as evidenced by accelerated water loss in detached *sllacs2* leaves compared to M82 controls (Fig. S5C and D). Quantitative analysis revealed a 50% water loss rate in mutant leaves compared to 35% in wild-type (Fig. S5E), suggesting that an intact trichome cuticle serves as a critical barrier regulating both volatile compound retention and water loss through the vapor phase, consistent with previous studies implicating trichomes in plant water relations [39, 40]. The maintenance of acylsugar content in *sllacs1* mutants contrasts with the reduced terpenoid levels observed in *sllacs2* mutants (Fig. S5A), suggesting that non-volatile acylsugars are less susceptible to loss through the compromised trichome barrier than volatile terpenoids.

To further explore the tissue-wide distribution of trichome barrier defects, we examined additional trichome-rich tissues in *sllacs2* mutants. TB staining analysis revealed stained type VI trichomes on young stems (Fig. S6A and B) and sepals (Fig. S6C), indicating that the role of SlLACS2 in maintaining trichome barrier function extends throughout the aerial portions of the plant.

### Glandular trichome-expressed *SlLACS1* and *SlLACS2* both activates long-chain fatty acids in the endoplasmic reticulum

**Figure 3:**
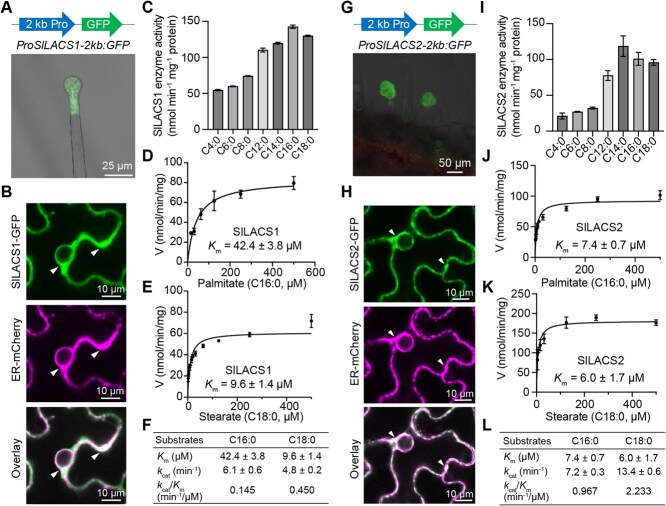
Type I/IV trichome-expressed SlLACS1 and type VI trichome-expressed SlLACS2 activate long-chain fatty acids in the endoplasmic reticulum (ER). A) Confocal fluorescence imaging showed that GFP fluorescence driven by *SlLACS1* 2 kb-length promoter is located in the tip cells of type I/IV trichomes. Long and arrows represent 2 kb-length *SlLACS1* promoter and GFP, respectively. Scale bar = 25 μm. B) Confocal microscopy revealed co-localization of C-terminal GFP-tagged SlLACS1 with the ER marker ER-mCherry in tobacco leaf epidermal cells. Arrows point to neutral grey signals from the merging of green and magenta fluorescence. Scale bars = 10 μm. C) Aliphatic fatty acids of different chain lengths were used as the substrates to test the long-chain acyl-CoA synthetase activity of SlLACS1. Mean amount of acyl-CoAs generated (nmol min^−1^ mg^−1^ proteins) was used to represent enzyme activities. The results are from three measurements ± SEM. D-E) The Michaelis–Menten plots were shown for SlLACS1 using the fatty acids palmitate (C16:0) D) and sterate (C18:0) E) as the substrates. F) Summary of SlLACS1 enzyme kinetic parameters using C16:0 and C18:0 as the substrates. G) Tissue-specific localization analysis revealed that *SlLACS2* is expressed in the tip cells of the type VI trichomes. GFP fluorescence was detected in the glandular heads of type VI trichomes in tomato plants stably transformed with *ProSlLACS2-2 kb:GFP-GUS*. Long and short arrows represent 2 kb-length *SlLACS2* promoter and GFP, respectively. Sclae bar = 50 μm. H) Confocal microscopy revealed co-localization of C-terminal GFP-tagged SlLACS2 with the ER marker ER-mCherry in tobacco leaf epidermal cells. Arrows point to neutral grey signals from the merging of green and magenta fluorescence. Scale bars = 10 μm. I) Aliphatic fatty acids of different chain lengths were used as the substrates to test the long-chain acyl-CoA synthetase activity of SlLACS2. Mean amount of acyl-CoAs generated (nmol min^−1^ mg^−1^ proteins) was used to represent enzyme activities. The results are from three measurements ± SEM. J-K) The Michaelis–Menten plots were shown for SlLACS2 when using the fatty acids palmitate (C16:0) J) and sterate (C18:0) K) as the substrates. L) Summary of SlLACS2 enzyme kinetic parameters using C16:0 and C18:0 as the substrates.

The disrupted structure and reduced composition of cuticle layers in type I/IV trichome tip cells of *sllacs1* mutant and type VI trichome tip cells of *sllacs2* mutant prompted us to examine their spatial expression patterns. Analysis of *ProSlLACS1-2 kb:GFP-GUS* transgenic plants revealed GFP fluorescence predominantly in type I/IV trichome tips (Fig. 3A). In contrast, transgenic tomato plants expressing either GFP-GUS or SlLACS2-GFP fusion proteins under control of a 2 kb *SlLACS2* promoter (*ProSlLACS2-2 kb:GFP-GUS* and *ProSlLACS2-2 kb:SlLACS2-GFP*) showed specific GFP expression in type VI trichome tip cells (Figs 3G and S7A). A truncated 786 bp promoter fragment (*ProSlLACS2-786 bp:GFP-GUS*) maintained this expression pattern (Fig. S7B), indicating that this region contains sufficient cis-regulatory elements for type VI trichome-specific expression. These distinct expression patterns align with their respective roles in cutin biosynthesis within type I/IV and type VI trichomes.

Subcellular localization studies revealed that both SlLACS1-GFP and SlLACS2-GFP fusion proteins co-localized with the ER-mCherry marker when transiently expressed in *Nicotiana benthamiana* leaves (Fig. 3B and H). This ER localization is consistent with previous findings that cutin monomer biosynthesis predominantly occurs in the ER [27], supporting their involvement in cutin biosynthesis. Biochemical characterization using His-tagged recombinant proteins purified from *Escherichia coli* (Fig. S8A–D) demonstrated that both SlLACS1 and SlLACS2 preferentially utilize long-chain fatty acids with chain lengths from C14 to C18 (Fig. 3C and I). This substrate preference aligns with their proposed roles in cuticle biosynthesis, as the activation of C16 and C18 long-chain fatty acids into long-chain acyl-CoA represents a crucial early step in cutin monomer biosynthesis [41].

Detailed enzyme kinetic studies further characterized the catalytic properties of the two enzymes. SlLACS2 exhibited high substrate affinity with a low *K*_m_ value of approximately 7 μM for both C16 and 6 μM for C18 fatty acids (Fig. 3J and K), with a notably higher catalytic efficiency (*k*_cat_/*K*_m_) for C18 compared to C16 (Fig. 3L). While SlLACS1 displayed higher *K*_m_ values and lower catalytic efficiency (*k*_cat_/*K*_m_) compared to SlLACS2 (Fig. 3C–F), both enzymes demonstrated substantial long-chain acyl-CoA synthetase activity. These biochemical properties, combined with their ER localization and trichome-specific expression patterns, strongly support the roles of SlLACS1 and SlLACS2 as long-chain acyl-CoA synthetases that activate C16 and C18 fatty acids to produce precursors for cutin monomer biosynthesis in their respective trichome types.

### *SlLACS1* and *SlLACS2* function redundantly in the epidermal cuticle biosynthesis of tomato leaf and fruit

**Figure 4:**
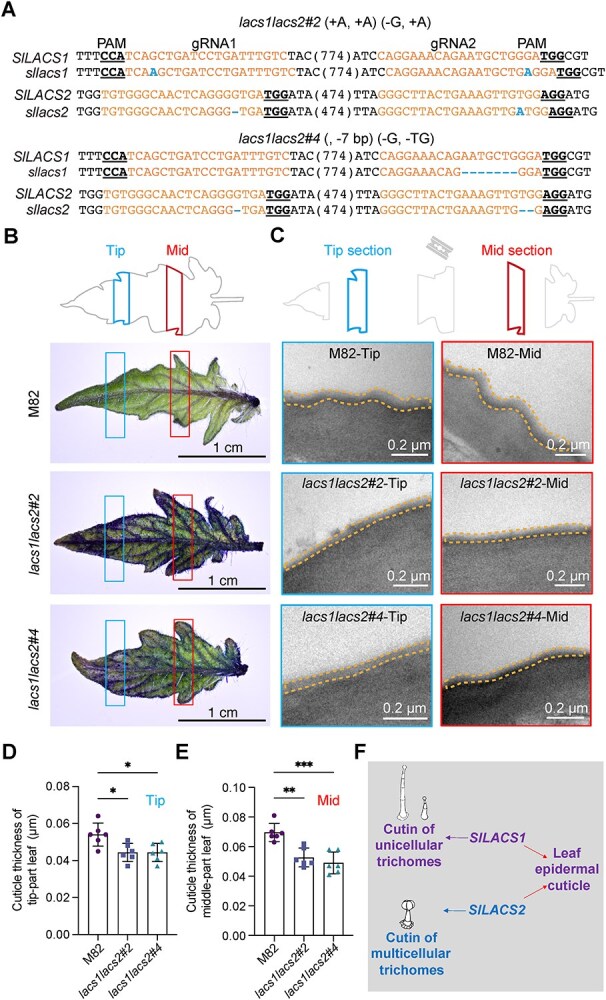
Redundant roles of *SlLACS1* and *SlLACS2* in leaf cuticle biosynthesis. A) Schematic illustration of generating *lacs1lacs2* double mutant using CRISPR-Cas9 technology. The orange letters indicate the gRNA target sites, with underlined letters showing the sites of the PAM. Resulting *SlLACS1* and *SlLACS2* mutations in the *lacs1lacs2#2* and *lacs1lacs2#4* mutants are denoted in dotted line or darker letters. B) Disrupted leaf epidermal permeability was observed in *lacs1lacs2* double mutant, caused by compromised leaf cuticle biosynthesis. In the left panel, the leaf TB staining experiments showed the entire leaves of *lacs1lacs2#2* and *lacs1lacs2#4* were stained but not the leaves of M82 control. For detailed analysis, the leaf tip (left rectangle) and middle (right rectangle) were examined using TEM. Scale bars = 1 cm. C) TEM images reveal a significant reduction in cuticle layer thickness in both leaf sections of the *lacs1lacs2* double mutants compared to M82. Scale bars = 0.2 μm. Dotted lines indicate the leaf epidermal cuticle layers. The thickness of leaf epidermal cuticle layers was quantified for the tip (left rectangle) D) and the middle (right rectangle) E) sections, with data derived from various slices from at least six leaves. Data are presented as means ± SD (*n* = 6); Unpaired *t*-test was performed and the significant differences are represented by black asterisks: ^*^  *P <* 0.05, ^**^  *P* < 0.01, ^***^  *P* < 0.001. F) A graphical summary illustrates the distinct and overlapping functions of *SlLACS1* and *SlLACS2* in cutin biosynthesis of trichomes and the leaf epidermis.

Initial characterization showed that single mutations in either *SlLACS1* or *SlLACS2* did not affect leaf epidermal permeability to TB (Figs 1 and S9). While our promoter-GUS analysis revealed enrichment of *SlLACS1* and *SlLACS2* in their respective trichome tip cells, comparative transcriptomic analysis of leaf epidermis (excluding trichomes) and mesophyll tissue revealed an unexpected finding: both genes are also expressed in epidermal cells (Fig. S10; Table S4). This broader expression pattern, combined with their similar biochemical activities in fatty acid activation, led us to hypothesize that SlLACS1 and SlLACS2 might functionally compensate for each other in maintaining epidermal cuticle integrity. To test this hypothesis, we generated *lacs1lacs2* double mutants in cultivated tomato M82 using CRISPR/Cas9 technology (Fig. 4A). Two independent homozygous lines, *lacs1lacs2#2* and *#4*, were isolated, each containing distinct deletions and insertions in both *SlLACS1* and *SlLACS2* coding sequences that are predicted to introduce premature stop codons (Fig. 4A).

Phenotypic analysis of *lacs1lacs2* double mutants revealed defects in both trichome and leaf epidermal barriers. TB staining showed increased permeability in both type I/IV and type VI glandular trichomes, recapitulating the phenotypes of their respective single mutants. Notably, both *lacs1lacs2#2* and *#4* exhibited pronounced dark blue staining on leaves, indicating compromised leaf epidermal barrier function (Fig. 4B). Correspondingly, TEM analysis confirmed thinner epidermal cuticle layers in these mutants compared to wild-type M82 controls (Fig. 4C–E), with consistent reduction observed in both tip and middle sections of the leaves (Fig. 4B–E). These findings demonstrate that while *SlLACS1* and *SlLACS2* have distinct roles in specific glandular trichome types, they function redundantly in maintaining leaf epidermal cuticle integrity (Fig. 4F).

**Figure 5:**
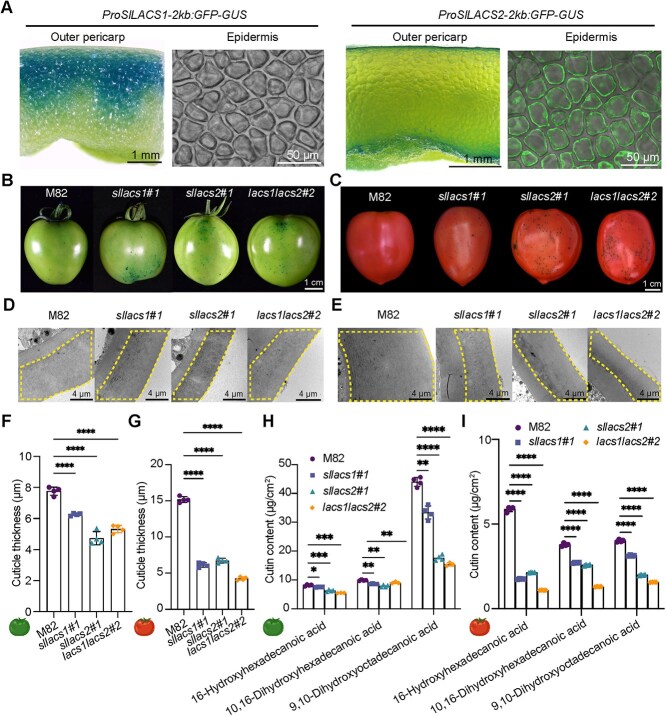
*SlLACS1* and *SlLACS2* are both involved in tomato fruit cuticle biosynthesis. A) Tissue expression pattern of *SlLACS1* and *SlLACS2* genes in tomato fruit. For the *ProSlLACS1-2 kb:GFP-GUS* tomato line, the GUS signal was observed in the mesocarp adjacent to the outer epidermis at MG stage, while no GFP signal was observed in the outer epidermis (left two panels). For the *ProSlLACS2-2 kb:GFP-GUS* tomato line, both GUS and GFP signals were observed predominantly in the outer and inner epidermis of MG stage fruit (right two panels). Scale bars = 1 mm (Outer pericarp) and 50 µm (Epidermis). B-C) Disrupted fruit epidermal permeability at the MG stage B) and RR stage C) was observed in the single and double mutants targeting *SlLACS1* and *SlLACS2*, as evidenced by the TB staining of the tomato fruits. Scale bars = 1 cm. D-E) TEM analysis demonstrates a reduction in cuticle layer thickness in the MG D) and RR E) stage fruit epidermis of *sllacs1#1*, *sllacs2#1*, and *lacs1lacs2#2* mutants compared to M82 control fruits, with dotted lines highlighting the cuticle layers. Scale bars = 4 μm. F-G) Cuticle thickness measurements of M82, *sllacs1#1*, *sllacs2#1*, and *lacs1lacs2#2* fruits at the MG F) and RR G) stages show statistically significant differences. The data, derived from various slices of at least four fruits, are presented as means ± SD (*n* = 4); Unpaired *t*-test was performed and significant differences are represented by black asterisks: ^****^  *P* < 0.0001**.** H-I) The cutin monomer composition of the fruit epidermis was quantified in M82, *sllacs1#1*, *sllacs2#1*, and *lacs1lacs2#2* fruits at the MG H) and RR I) stages. Data are presented as means ± SD (*n* = 4); Unpaired *t*-test was performed and significant differences are represented by black asterisks: ^*^  *P <* 0.05, ^**^  *P* < 0.01, ^***^  *P* < 0.001, ^****^  *P* < 0.0001.

Analysis of the TEA database [42] revealed that both genes are expressed in fruit outer and inner epidermis, with expression levels peaking during the fruit expansion phase—from 10 days post-anthesis (DPA) to the mature green (MG) stage—and subsequently declining to minimal levels by the red ripening (RR) stage (Fig. S11). This expression pattern suggested potential roles for these genes in fruit development. Indeed, during post-harvest storage, we observed accelerated wilting in *sllacs2* mutant fruits (Fig. S12), indicating possible alterations in fruit water retention properties. To further understand their roles in fruit development, we examined GUS and GFP signals in the fruits of *ProSlLACS1-2 kb:GFP-GUS* and *ProSlLACS2-2 kb:GFP-GUS* tomato lines. Consistent with the transcriptome data, both genes showed fruit-specific expression patterns: *SlLACS1* predominantly in the mesocarp adjacent to the outer epidermis, and *SlLACS2* in both outer and inner epidermis (Figs 5A and S11).

To examine the roles of *SlLACS1* and *SlLACS2* in fruit cutin biosynthesis, we analyzed fruit cuticle integrity in single (*sllacs1* and *sllacs2*) and double (*lacs1lacs2*) mutants. All mutants exhibited enhanced TB permeability at both MG and RR stages, indicating compromised cuticular barriers (Fig. 5B and C). TEM analysis confirmed significant reductions in cuticle thickness in mutants compared to M82 controls (Fig. 5D–G). GC/MS analysis revealed substantial decreases in specific cutin monomers—16-hydroxyhexadecanoic acid, 10,16-dihydroxyhexadecanoic acid, and 9,10-dihydroxyoctadecanoic acid—in both single and double mutants (Fig. 5H and I). A comparative evaluation of single versus double mutants revealed that at the RR stage—when *SlLACS1* and *SlLACS2* expression are minimal—cuticle thickness and cutin monomer levels were notably lower in the *lacs1lacs2* mutant than in *sllacs1* or *sllacs2* (Fig. 5G and I), indicating functional redundancy of the two genes. Conversely, at the MG stage, when *SlLACS1* and *SlLACS2* expression are high, the cuticle morphology and monomer composition disparity between single and double mutants was less pronounced (Fig. 5F and H). This difference likely reflects active cutin biosynthesis during the MG stage, whereas the RR stage represents the culmination of cutin development.

### *SlLACS1* and *SlLACS2* redundantly control pollen viability through pollen coat formation

**Figure 6:**
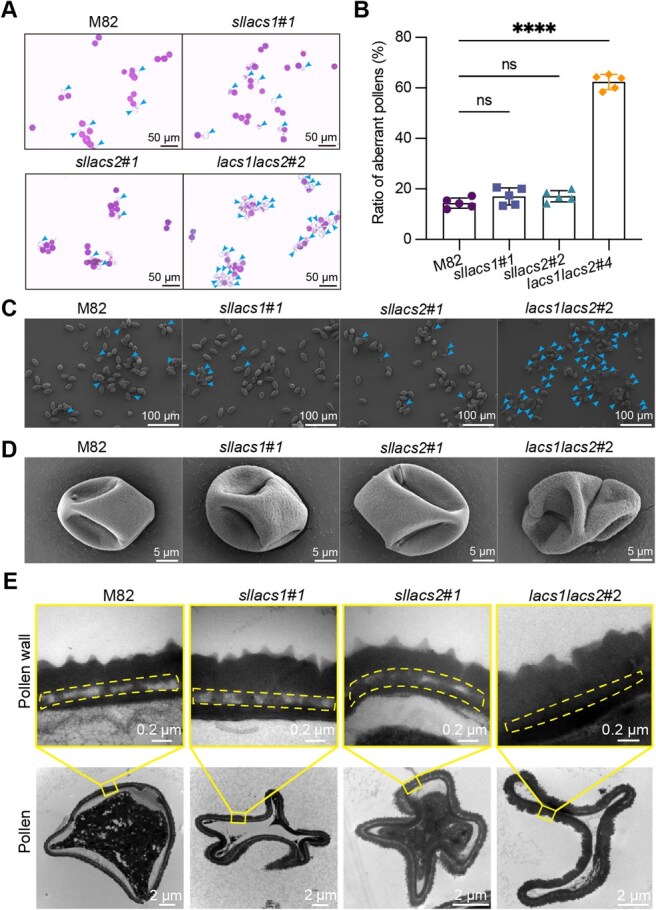
*SlLACS1* and *SlLACS2* redundantly affect tomato pollen viability through participating in pollen coat formation. A) Alexander staining of pollens from flowers of M82, *sllacs1*#*1*, *sllacs2*#*1*, and *lacs1lacs2*#*2* plants, with viable pollen stained red-purple and non-viable or aberrant pollen unstained, as indicated by arrows. Scale bars = 50 μm. B) Percentage of abnormal pollen in mature anthers of M82, *sllacs1#1*, *sllacs2#1*, and *lacs1lacs2#2* plants. Values represent means ± SD (*n* = 5); statistical significance determined by unpaired *t*-test (^****^  *P* < 0.0001; ns, *P* > 0.05). C) Scanning electron microscopy (SEM) provides a comparative overview of pollen morphology from the same genotypes, highlighting aberrant pollen in the mutants, especially pronounced in the double mutant as indicated by arrows. Scale bars = 100 μm. D) SEM reveals detailed morphological defects in aberrant pollen grains from each genotype, with the most noticeable deformities in the *lacs1lacs2#2* pollen. Scale bars = 5 μm. E) TEM cross-sections of aberrant (second row) pollen grains reveal structural differences, with particular attention to the pollen coat. Scale bars = 2 μm. Enlarged images of the pollen wall (top row) illustrate the pollen coat structure (delineated by dashed lines), which appears compromised in the *lacs1lacs2#2* mutant pollen. Scale bars = 0.2 μm.

During phenotypic characterization of the mutant lines, we observed premature flower withering and reduced fruit set in *lacs1lacs2* double mutants. To investigate whether this reproductive defect might be related to LACS gene function in floral organs, we examined the expression pattern in reproductive tissues using our established *ProSlLACS2-2 kb:GFP-GUS* reporter lines. Strong GUS signals were detected in both pollen and tapetum (Fig. S13), suggesting potential roles for LACS genes in male reproductive development. This finding led us to examine the pollen viability in both single and double mutants. Alexander staining revealed that while single mutants and wild-type M82 maintained over 80% viable pollen, the *lacs1lacs2* double mutant exhibited a significant reduction to 37.6% viability (Fig. 6A–C). To further characterize this pollen defect, we performed scanning electron microscope (SEM) analysis. While normal pollen appeared similar across all genotypes (Fig. S14A), abnormal pollen grains in the double mutant showed more severe structural distortions compared to single mutants or M82 controls (Fig. 6D). These results suggest that SlLACS1 and SlLACS2 play redundant roles in maintaining pollen viability, likely through their functions affecting the pollen development.

To elucidate the cause of deficient pollen structures in the *lacs1lacs2* double mutant, we examined the ultrastructure of normal and abnormal pollen grains using TEM. Our findings indicated alterations in the pollen coat layer of the *lacs1lacs2* mutant. Pollen coat, a hydrophobic layer composed of fatty acids and derivatives secreted by tapetum, is essential for pollen functionality [43, 44]. In the normal pollen, the pollen coat appears semi-transparent under TEM, filling the interstices within the pollen wall (Fig. S14B). While this layer remained intact in abnormal pollen of single mutants and M82 controls, it was notably absent in abnormal pollen of the *lacs1lacs2* double mutant (Fig. 6E). Further analysis by GC/MS revealed significant reductions in palmitic acid and α-linolenic acid in the *lacs1lacs2* pollens (Fig. S15)*.* These findings demonstrate that SlLACS1 and SlLACS2 function redundantly in tapetum pollen coat biosynthesis, with their combined loss compromising pollen structural integrity.

### Evolutionary duplication and functional diversification of LACS1 and LACS2 coincide with plant terrestrialization

Our findings demonstrate that SlLACS1 and SlLACS2 are indispensable for epidermal cuticle synthesis across diverse tomato tissues and are crucial for maintaining water relations in leaves and fruits. This led us to hypothesize that LACS1 and LACS2 genes may have evolved as a critical innovation during plant terrestrialization, enabling the development of protective hydrophobic barriers essential for survival in a terrestrial environment.

**Figure 7:**
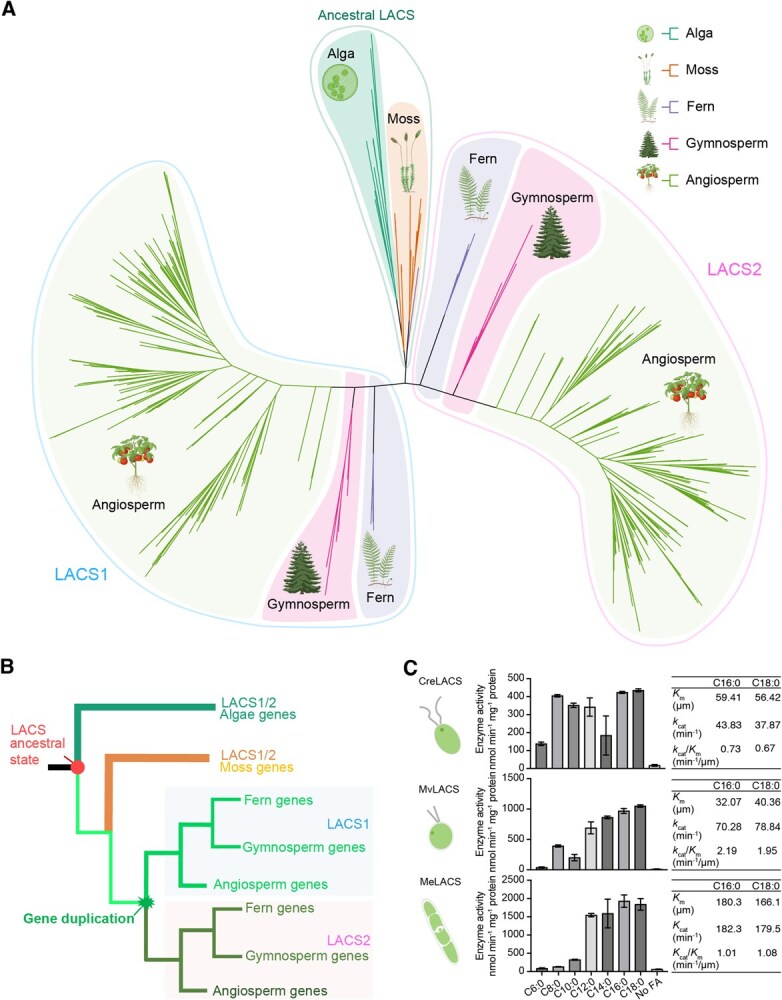
Phylogenetic and biochemical analysis of LACS1/2 in green plants. A) A phylogenetic tree composed of 402 LACS1/2 homologs from 220 green plant species ranging from algae to flowering plants is presented. The tree was constructed using the maximum likelihood (ML) method with details provided in the experimental section. Branches are color-coded to represent different plant groups: algae (light seagreen background), moss (light orange background), fern (light purple background), gymnosperm (light pink background), and angiosperm (bright green background). The evolution of distinct ancestral LACS, LACS1, and LACS2 clades is circled by the lines shown in the middle, left, and right, respectively. B) The phylogenetic trajectory illustrates the divergence of LACS1 and LACS2 throughout plant evolution, tracing their lineage across algal, moss, fern, gymnosperm, and angiosperm groups. C) Enzymatic analysis of algal LACS1/2 homologs to infer its ancestral biochemical function. Fatty acids of different chain lengths were used as the substrates to test the enzyme activity of three algal LACSs (CreLACS, MvLACS, MeLACS). The enzyme activity was represented by the mean amount of acyl-CoAs generated (nmol min^−1^ mg^−1^ proteins). The parameters of enzyme kinetics of CreLACS, MvLACS, and MeLACS for C16 and C18 fatty acid substrates were shown in the tables on the right. Cre, *Chlamydomonas reinhardtii*; Mv, *Mesostigma viride*; and Me, *Mesotaenium endlicherianum*.

To elucidate the evolutionary history of LACS1 and LACS2 within the plant kingdom, we conducted a phylogenetic analysis of 402 homologs from 220 species, spanning from green algae to terrestrial plants (Tables S5 and S6). This analysis identified three principal lineages, categorized into LACS1 homologs (Fig. 7A, left irregular circle), LACS2 homologs (Fig. 7A, right irregular circle), and the ancestral LACS (Fig. 7A, middle irregular circle). Consistent with the hypothesis that LACS gene diversification accompanied plant terrestrialization, we found that LACS1 and LACS2 are exclusive to ferns, gymnosperms, and angiosperms, whereas ancestral LACS are primarily associated with algae and early evolving land plants (Fig. 7A). Phylogenetic evidence suggests that the ancestral gene of LACS1 and LACS2 emerged in the common ancestor of green plants (Fig. 7B). A subsequent duplication event in the common ancestor of ferns, gymnosperms, and angiosperms gave rise to LACS1 and LACS2 (Fig. 7B). This gene duplication coincided with plant terrestrialization, likely enabling the development of specialized hydrophobic barriers necessary for survival in a terrestrial environment.

To understand the evolutionary trajectory of LACS1/2 enzymatic function, we investigated whether the ability to convert long-chain fatty acids to acyl-CoAs was conserved from algal ancestors or emerged in land plants. We isolated LACS homologs from three basal green plant species: *Chlamydomonas reinhardtii*, *Mesostigma viride*, and *Mesotaenium endlicherianum*. Enzymatic assays of their purified recombinant proteins (Fig. S16) revealed that all three algal homologs—CreLACS, MvLACS, and MeLACS—preferentially utilize C12 to C18 fatty acids as substrates (Fig. 7C), similar to SlLACS1 and SlLACS2 (Fig. 3C and I).

Detailed enzyme kinetic analyses showed varying substrate affinities among the algal LACS enzymes. MvLACS exhibited the highest substrate affinity, with low *K*_m_ values of approximately 32 μM for C16 and 40 μM for C18, and superior catalytic efficiency (*k*_cat_/*K*_m_) of 2.19 and 1.95 min^−1^ μM^−1^ for C16 and C18 fatty acids, respectively. MeLACS showed lower substrate affinity with higher *K*_m_ values (approximately 180 μM for C16 and 166 μM for C18) but maintained higher catalytic efficiency than CreLACS. CreLACS demonstrated the lowest catalytic efficiency among the three enzymes (Figs 7C and S17). These results reveal that while the substrate affinity and catalytic efficiency of algal LACS enzymes vary, the ability to synthesize long-chain acyl-CoAs from C16 and C18 fatty acids is a conserved ancestral function, highlighting the evolutionary foundation for the roles of LACS in plant lipid metabolism.

## Discussion

### LACS gene duplication and the rise of tissue complexity in land plants

**Figure 8:**
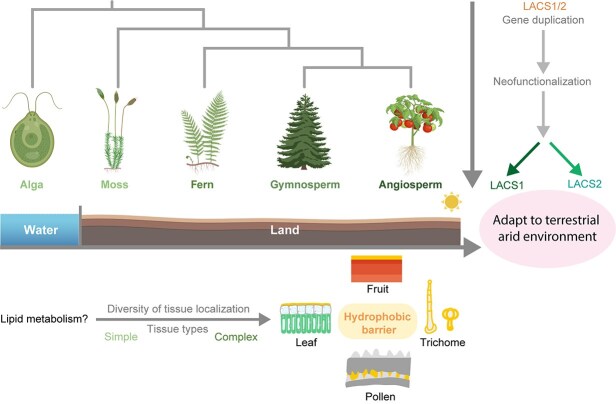
Evolutionary duplication and functional diversification of LACS1 and LACS2 coincide with plant terrestrialization. Phylogenetic analyses indicate the progenitor gene of LACS1 and LACS2 originated in the last common ancestor of extant green plants. Subsequent gene duplication and divergence occurred, with the pivotal LACS1 and LACS2 duplication event traced back to a common ancestor of ferns, gymnosperms, and angiosperms. This duplication is posited to have occurred concomitantly with plant terrestrialization, suggesting a role for these genes in enhancing hydrophobic barrier synthesis, which facilitated the development of complex terrestrial structures such as leaves, fruits, trichomes, and pollen coat in angiosperms, thereby contributing to the adaptation of plants to dry terrestrial environments.

Our findings reveal that LACS1/2 enzymes have maintained their fundamental biochemical function—catalyzing the conversion of medium-to-long chain fatty acids to acyl-CoA—throughout plant evolution. However, while these enzymes might serve alternative roles in algal lipid metabolism, their duplication and subsequent neofunctionalization during plant terrestrialization appear driven by tissue-specific expression diversification rather than changes in catalytic activity (Fig. 8). This evolutionary trajectory aligns with the increasing demands for tissue complexity and survival in terrestrial environments. SILACS1 and SILACS2 play essential roles in cuticle formation across diverse tomato tissues, exhibiting both specialized functions in distinct trichome types and redundant roles in leaf and fruit cuticle integrity. While Wu *et al.* [45] recently characterized SlLACS1's role in tomato cuticular wax biosynthesis, our work reveals previously unrecognized spatial specialization: SlLACS1 specifically maintains cuticle integrity in type I/IV trichomes, while SlLACS2 governs type VI trichome barriers. Crucially, we demonstrate functional redundancy between these paralogs in pollen coat formation that ensuring reproductive viability. The functional redundancy observed between *SlLACS1* and *SlLACS2* in certain tissues, coupled with their distinct tissue-specific expression patterns in others, points towards an intriguing evolutionary trajectory. Our phylogenetic analysis suggests that LACS1 and LACS2 homologs are exclusive to land plants (ferns, gymnosperms, and angiosperms), emerging after the divergence from aquatic ancestors (Fig. 7A). This timing corresponds with the increased tissue complexity accompanying terrestrialization and the concurrent need for sophisticated hydrophobic barriers, as evidenced by fossil records and comparative genomics [7, 46].

The ancestral LACS, predominantly found in algae and basal land plants, likely played a role in general lipid metabolism, as demonstrated by the conserved substrate specificity of algal LACS homologs to utilize medium-to-long-chain fatty acids (Fig. 7C). Terrestrial colonization created selective pressure for hydrophobic barriers to prevent desiccation and UV damage, driving LACS gene duplication and specialization for cuticle biosynthesis. The evolution of novel plant structures—leaves, stems, and reproductive organs—necessitated tissue-specific protective mechanisms, fulfilled through LACS1 and LACS2 specialization in angiosperms.

While they exhibit functional redundancy in some tissues, such as the leaf epidermis and pollen, their distinct expression patterns in other tissues, such as specific trichome types and fruit layers, suggest a refinement of function tailored to specific ecological and developmental requirements. For instance, their distinct expression patterns in different trichome types—SlLACS1 in type I/IV trichomes producing acylsugars, and SlLACS2 in type VI trichomes synthesizing volatile terpenoids—reflect their functional specialization, which likely evolved in response to the distinct ecological roles and metabolic demands of these trichomes. The presence of less specialized LACS homologs in early-diverging land plants like mosses supports the stepwise evolution of these genes alongside increasing plant complexity [47]. Moreover, the functional redundancy of *SlLACS1* and *SlLACS2* observed in leaf and fruit cuticle formation, as well as in pollen coat biosynthesis, may serve as an evolutionary buffer, increasing the robustness of cuticle development against mutations. This genetic robustness could be particularly advantageous in the face of fluctuating environmental conditions, ensuring the consistent production of this critical protective barrier.

### Trichome-specific cuticle biosynthesis mediated by *SlLACS1* and *SlLACS2*

While cuticle research has traditionally focused on leaf epidermis and fruit surfaces [48], our study reveals distinct cuticle layers on both type I/IV and type VI glandular trichomes in tomato. Through a combination of phenotypic characterization (Figs 1 and S6), TEM imaging (Fig. 2), and cutin monomer analysis (Fig. 2), we demonstrate that the plant aerial surface possesses a complete hydrophobic barrier encompassing not just the epidermis, but also trichomes. The tissue-specific roles of SlLACS1 and SlLACS2 in trichome cuticle formation exemplify how gene duplication enabled specialized barrier function in different plant structures. The distinctive phenotypes of our mutants—increased permeability specifically in type I/IV trichomes for *sllacs1* and type VI trichomes for *sllacs2*—demonstrate precise spatial control of cuticle biosynthesis in specialized structures.

This specialized regulation has significant functional implications. Glandular trichomes serve crucial roles in plant defense against herbivores and pathogens [19], and our finding that compromised type VI trichome cuticle leads to reduced terpenoid accumulation suggests that the cuticle plays a crucial role in volatile compound retention. Volatile terpenoids, synthesized in the glandular trichome cells, must traverse several cellular layers—the cytoplasm, cell membrane, cell wall, and finally, the cuticle—before being released into the atmosphere [49]. The cuticle, as the outermost layer, presents a significant barrier to volatile diffusion [50]. The thinner cuticle observed in *sllacs2* mutants likely reduces this barrier, facilitating the release of volatile terpenoids and explaining the observed reduction in their accumulation in leaves. This modulation of volatile emission by the cuticle is likely crucial for regulating plant-insect interactions and other ecological processes. The evolution of distinct cuticle regulation in different trichome types appears to parallel their specialized functions, influencing their interactions with water and other substances [51, 52]. The maintenance of normal acylsugar levels in *sllacs1* mutants, despite compromised type I/IV trichome cuticle, highlights the resilience of non-volatile defensive compounds to barrier disruption, which contrasts with the increased loss observed for volatile compounds.

Our results reveal broader implications for plant–environment interactions. Trichome density and morphology affect various leaf properties, including gas diffusion and light capture [53, 54]. The accelerated water loss in *sllacs2* mutants demonstrates that trichome cuticle integrity contributes significantly to whole-plant water balance, extending beyond the traditional view of epidermal cuticle function. These observations suggest that trichome cuticles evolved not only for specialized metabolite retention but also as integral components of plant water management systems. The presence of cuticle on trichomes likely modulates multiple aspects of plant-environment interactions, from volatile organic compounds emission to water relations and stress tolerance. This structural adaptation appears to serve multiple functions: protecting specialized metabolite-producing cells, preventing compound release, and contributing to overall plant water homeostasis. The distribution and properties of trichome cuticles may also influence the retention of atmospheric water and pollutants on the leaf surface, suggesting additional roles in plant environmental adaptation that require further investigation.

### *SlLACS1* and *SlLACS2* contribute to both cuticle and pollen coat biosynthesis

Our study showed that SlLACS1 and SlLACS2 exhibit redundant function in forming the leaf epidermal cuticle and fruit cuticle. Intriguingly, it also reveals that this functional overlap extends to male reproductive development, specifically in the formation of pollen coat, a specialized hydrophobic layer that coats pollen grains. While structurally distinct from the cuticle, pollen coat serves a similar purpose: preventing desiccation and ensuring pollen viability during pollination [43]. The expression of *SlLACS2* in the tapetum (Fig. S13), where pollen coat is synthesized and secreted [55], suggests its involvement in pollen coat production. The lack of pollen viability defects in *sllacs1* or s*llacs2* single mutants, contrasted with the severe reduction in viable pollen and aberrant pollen morphology in the *lacs1lacs2* double mutant (Fig. 6), indicates functional redundancy between these genes in maintaining pollen development and viability. Given the similar biochemical functions of SlLACS1 and SlLACS2 demonstrated in our study, and their redundant roles in cuticle formation, we propose that these enzymes work cooperatively in tapetal cells to support pollen coat biosynthesis, thereby maintaining pollen viability.

This dual function of LACS enzymes in synthesizing distinct hydrophobic barriers reveals their fundamental importance in plant survival and reproduction. Despite compositional differences between pollen coat—a complex mixture of lipids, pigments, and aromatic compounds—and cuticle, which consists of cutin polymer and waxes, both barriers require fatty acid precursors activated by LACS enzymes [56]. The ability of SlLACS1 and SlLACS2 to contribute to both barriers emphasizes their central role in establishing hydrophobic protective layers across diverse plant tissues and developmental stages. While our study focused primarily on the cutin component of the cuticle, the involvement of SlLACS1/2 in activating long-chain fatty acids suggests they also contribute to the biosynthesis of cuticular waxes. This is supported by a recent study showing altered wax composition in *sllacs1* mutants [45]. Future research will address this by characterizing wax profiles in leaves and fruit of the *lacs1lacs2* double mutant. The analysis of surface wax, combined with physiological assays of cuticle properties, will provide a more complete understanding of how SlLACS1/2 contribute to the overall structure and function of the plant cuticle barrier.

The involvement of these genes in tapetum pollen coat biosynthesis raises evolutionary questions about pathway recruitment and diversification. The presence of LACS homologs in basal land plants lacking pollen, such as ferns, provides an opportunity to investigate the evolutionary sequence of LACS recruitment for different barrier types. Such comparative studies could illuminate the diverse forms and functions of pollen coat observed across seed plants [57] and reveal how these protective structures evolved during plant terrestrialization.

In summary, our findings support a model where LACS gene duplication and functional diversification facilitated the evolution of tissue complexity and enabled successful plant adaptation to terrestrial environments (Fig. 8). This evolutionary trajectory shows how gene duplication, combined with changes in where and when genes are expressed in specific tissues, can lead to new morphological traits—even without changes to the enzymatic functions. Further investigation of LACS functional diversity across plant lineages will enhance our understanding of how these genes contributed to the remarkable diversity of hydrophobic barriers in the plant kingdom.

## Materials and methods

### Plant materials and growth conditions

Tomato (*Solanum lycopersicum* cv M82) seeds were obtained from the C.M. Rick Tomato Genetic Resource Center (https://tgrc.ucdavis.edu/) and served as the wild type (WT) in this study. Seeds were germinated on moistened filter paper at room temperature before transplanting to 32-cell plastic flats. Plants were grown in a growth chamber at 60% relative humidity under a photoperiod of 14 h light (500 μmol m^−2^ s^−1^) at 25°C and 10 h dark at 20°C. Fruits were tagged at 10 days post-anthesis (DPA) and harvested at MG (35 DPA) and RR (54 DPA) developmental stages.

### RNA extraction, sequencing, and WGCNA analysis

Epidermal tissues were dissected from young leaves of four-week-old M82 plants under a dissection microscope. Using ultrafine forceps, we collected epidermis with attached trichomes from both adaxial and abaxial epidermis with trichomes (EPT). The exposed mesophyll was harvested from the adaxial side in regions not covered by abaxial EPT, while the remaining upper epidermi with a combination of adaxial EPT and mesophyll was collected separately. The trichome-free leaf epidermis was obtained by carefully removing all types of trichomes from the tomato leaf adaxial surface with ultrafine forceps, followed by gentle peeling of the remaining epidermal layer. Three biological replicates were used for each set of samples. All samples were collected avoiding major leaf veins. Total RNA was extracted from the dissected tissues (adaxial EPT, abaxial EPT, mesophyll, upper epidermis, and trichome-free leaf epidermis) using TRIzol reagent (Invitrogen, CA, USA). RNA quality and quantity were assessed using a NanoDrop 2000 spectrophotometer (Thermo Fisher Scientific, MA, USA) and Agilent 2100 Bioanalyzer (Agilent Technologies, CA, USA). Libraries were prepared using a VAHTS Universal V6 RNA-seq Library Prep Kit (Vazyme, Nanjing, China) and sequenced on an Illumina NovaSeq 6000 platform (Illumina, USA). After removing adapter sequences and low-quality reads using fastqc (version 0.12.0), clean reads were mapped to the tomato reference genome (Heinz1706, build 3.00) using HISAT2 (version 2.2.1) [58]. Gene expression levels were quantified as fragments per kilobase of transcript per million mapped reads (FPKM) using StringTie v2.1.4 [59]. Differentially expressed genes (DEGs) were identified using edgeR v3.38.4, with |log2FoldChange| > 1 and adjusted *P*-value <0.05 as thresholds. To identify clusters of genes that exhibit similar expression patterns across the various dissected leaf tissues, we generated a coexpression network using the WGCNA package in R [60, 61], with the following settings: soft-thresholding power = 10, minimum module size = 30, max module size = 5000, branch merge cut height = 0.25. The analysis yielded 38 gene modules with distinct expression profiles. The raw sequence data of RNA-seq has been deposited in the Sequence Read Archive in National Center for Biotechnology Information, under the accession number PRJNA1185636.

### Virus-induced gene silencing assay

Tobacco rattle virus (TRV)-based vectors pTRV1 and pTRV2-LIC were employed for virus-induced gene silencing (VIGS) experiments [62, 63]. Target gene fragments were designed using the Solanaceae Genomics Network VIGS tool (http://vigs.solgenomics.net/) and inserted into the pTRV2-LIC vector. *Agrobacterium tumefaciens* strain GV3101 containing pTRV1, pTRV2 constructs, or empty pTRV2 was cultured overnight at 28°C in media supplemented with kanamycin (50 µg mL^−1^) and gentamicin (10 µg mL^−1^). Bacterial cells were harvested by centrifugation (8000 g, 5 min, 4°C), washed, and resuspended in infiltration buffer (10 mM MES pH 5.5, 10 mM MgCl_2_, 200 μM acetosyringone). After 3 h incubation at room temperature, pTRV2 cultures were mixed with equal volumes of pTRV1 culture (final OD_600_ = 1) and infiltrated into tomato sprouts following established protocols [64]. Silencing of the phytoene desaturase (PDS) gene served as a positive control, and phenotypic analysis of target gene-silenced plants was conducted upon observation of photo-bleaching in PDS-silenced plants. Primer sequences are provided in Table S7.

### Constructs and generation of transgenic plants

CRISPR/Cas9-mediated knockout lines of *SlLACS1*, *SlLACS2*, and *LACS1LACS2* were generated following established protocols [65]. sgRNAs were selected using CRISPR-P 2.0 (http://crispr.hzau.edu.cn/CRISPR2/) [66], and corresponding primers were designed according to multi-gRNA array assembly protocols [65]. The resulting PCR fragments were cloned into binary vector pDIRECT_21C to generate *SlLACS1_21C*, *SlLACS2_21C*, and *LACS1LACS2_21C* constructs. For tissue localization studies, 2 kb promoter regions of *SlLACS1* and *SlLACS2*, and a 786 bp promoter fragment of *SlLACS2* were amplified and cloned into pKGWFS7 via GATEWAY cloning, yielding *ProSlLACS1-2 kb:GFP-GUS*, *ProSlLACS2-2 kb:GFP-GUS*, and *ProSlLACS2-786 bp:GFP-GUS* plasmids. The *ProSlLACS2-2 kb:SlLACS2-GFP* construct was generated by amplifying and assembling a 2 kb *SlLACS2* promoter fragment, *SlLACS2* genomic sequence, and GFP into pENTR using Golden Gate assembly, followed by GATEWAY cloning into pK7WG. All constructs were transformed into tomato M82 via *A. tumefaciens* (GV3101)-mediated transformation [67, 68]. Primer sequences are provided in Table S7.

### Acylsugar analysis

Leaf surface acylsugars were extracted following the protocol available at Protocols.io (http://dx.doi.org/10.17504/protocols.io.xj2fkqe). Briefly, the youngest fully developed leaves were extracted with 1 ml of acetonitrile/isopropanol/water (3:3:2) containing 0.1% formic acid and 1 μM telmisartan (internal standard). Samples were gently agitated for 2 min, and the extraction solvent was collected and stored in glass vials at −20°C until analysis. Metabolite profiling was performed using an Agilent 1290 Infinity II UHPLC system coupled to an Agilent 6545B Q-TOF mass spectrometer (Agilent Technologies) with an electrospray ionization (ESI) probe in negative mode. The Q-TOF parameters were: gas temperature, 275°C; gas flow, 8 L/min; nebulizer pressure, 35 psig; sheath gas temperature, 300°C; sheath gas flow, 11 L/min; capillary voltage, 3500 V; nozzle voltage, 0 V; fragmentor voltage, 150 V; skimmer voltage, 65 V. Samples (5 μl) were separated on a C18 column at 40°C using a 21-min binary gradient of water (A) and acetonitrile (B) at 0.3 ml/min: 5%–60% B (0–3 min), 60%–100% B (3–15 min), 100% B (15–18 min), 100%–5% B (18–18.1 min), and 5% B (18.1–21 min). Data were acquired in Auto MS/MS mode (m/z 50–1700) and analyzed using Agilent MassHunter Workstation software (version 10.0).

### Protein subcellular localization in tobacco mesophyll cells

For subcellular localization analysis, full-length coding sequences of *SlLACS1* and *SlLACS2* (without stop codons) were PCR-amplified from M82 leaf cDNA using primers listed in Table S7. The amplified sequences were cloned into *Pro35S:GFP* binary vectors to generate *Pro35S:SlLACS1-GFP* and *Pro35S:SlLACS2-GFP* constructs using ClonExpress II One Step Cloning Kit (Vazyme, China). The resulting plasmids were transformed into *A. tumefaciens* strain GV3101. Transient expression was performed following previous protocols with modifications [69]. Briefly, transformed *A. tumefaciens* cells were washed and resuspended in infiltration buffer (20 mM acetosyringone, 50 mM MES pH 5.7, 0.5% glucose [w/v], 2 mM Na_3_PO_4_) to OD_600_ = 0.05. Four-week-old *Nicotiana benthamiana* plants grown under short-day conditions (8 h light, 21°C) were infiltrated and maintained under the same conditions for three days before imaging. GV3101 containing ER-mCherry marker was co-infiltrated as an ER-localization control.

### Confocal microscopy

Imaging was performed using a Nikon A1 laser scanning confocal microscope with Nikon NIS-Elements Advanced Research software. GFP fluorescence in tomato transformant trichomes and tobacco mesophyll cells was visualized using 488 nm excitation and 505–525 nm emission filters. mCherry fluorescence in tobacco mesophyll cells was detected using 561 nm excitation and 580–630 nm emission filters.

### Cuticle analysis by the TEM

Pollen, leaves, and fruit discs were fixed in 2.5% glutaraldehyde in 0.1 M phosphate buffer (PB, pH 7.0) for 8 h at 4°C, followed by three 15-min PB rinses. Samples were post-fixed with 1% OsO_4_ in PB for 2 h at room temperature (RT) and washed again in PB for 15 min. Dehydration was performed through graded ethanol series (30%, 50%, 70%, 80%; 15 min each) followed by acetone series (90%, 95%, 100% twice; 15–20 min each). Samples were infiltrated with increasing concentrations of resin in acetone (50% for 1 h, 66% for 3 h, 100% for 10 h) at RT, then embedded in pure resin and polymerized at 70°C for 12 h. Ultrathin sections were prepared using a Leica EM UC7 ultramicrotome, stained with uranyl acetate and alkaline lead citrate (5–10 min each), and examined using a Hitachi Model H-7650 TEM.

### Protein expression and LACS enzyme assay

For heterologous expression of His-tagged SlLACS1, SlLACS2, CrLACS (Cre13.g566650.t1.2), MeLACS (ME000128S00185), and MvLACS (Mv16422-RA.1) proteins, full-length ORFs were PCR-amplified using specific primers (Table S7) and cloned into *BamH*I/*Sal*I-linearized pET28b vector using One Step Infusion Mix (TOROIVD). The resulting constructs were transformed into *E. coli* Rosetta 2 (DE3). Protein expression was induced with 0.05 mM isopropyl *β*-D-1-thiogalactopyranoside (IPTG) at OD_600_ = 0.5, and cultures were incubated overnight at 16°C with 120 rpm shaking. His-tagged proteins were purified using Ni-NTA agarose (Smart-Lifesciences, cat. no. SA004025) following manufacturer's instructions. Long-chain acyl-CoA synthetase activity was measured using a coupled enzyme assay as described previously [70]. In brief, enzymatic assays were performed using a SpectraMax iD3 multimode plate reader. Fatty acid substrates (5 mM stock) were prepared in 5% Triton X-100. The reaction mixture contained 0.1 M Tris–HCl (pH 7.5), 2 mM DTT, 5 mM ATP, 10 mM MgCl_2_, 0.5 mM CoA, 0.8 mM NADH, 250 µM fatty acid substrate, 1 mM phosphoenolpyruvate, and coupling enzymes (20 U myokinase, 10 U each of pyruvate kinase and lactate dehydrogenase). Reactions were initiated by adding 5 μL protein (1–2 μg) to 95 μL premix in 96-well plates. NADH oxidation was monitored at 340 nm using the NADH extinction 6.22 cm^2^ μmol^−1^ for every 1 min for 30 min at 30°C. For kinetic analysis, fatty acid concentrations were varied from 0 to 1 mM with NADH set at 1 mM. Acyl-CoA production was calculated based on a 2:1 stoichiometry of NADH oxidation. Fatty acid substrates included sodium butyrate (C4:0), sodium hexanoate (C6:0), sodium octanoate (C8:0), sodium decanoate (C10:0), sodium laurate (C12:0), sodium myristate (C14:0), sodium palmitate (C16:0), and sodium stearate (C18:0) (all from Sigma-Aldrich).

### GC–MS analysis of leaf terpenoids

Volatile terpenes were analyzed from leaflets of the second-youngest leaf of four-week-old plants. Compounds were extracted as previously reported [21] and analyzed using a 7890B GC system equipped with 5977B GC/MSD detector (Agilent Technologies). Samples (5 μL) were separated on a DB-5MS + DG column (30 m × 0.25 mm, 0.25 μm film; Agilent Technologies) using previously established parameters [21]. Metabolites were identified by comparing mass spectra with the NIST GC library (version 17, https://chemdata.nist.gov/dokuwiki/doku.php?id=chemdata:nistlibs), and peak areas were integrated using MassHunter software (Agilent Technologies). Compound quantities were normalized to tetradecane internal standard and leaf dry weight.

### Water loss rate assay

Water loss was measured in fully expanded leaves from four-week-old tomato plants. Fresh weights were recorded immediately after detachment, and leaves were maintained at room temperature with periodic weight measurements. Water loss rate was calculated as (FW - Wt) × 100%, where FW represents initial fresh weight and Wt represents leaf weight at each time point post-detachment.

### Cutin monomer analysis

Cutin monomer analysis was performed following modified protocols [71, 72]. For trichome analysis, type VI trichome glandular heads were collected from young leaves of two-month-old seedlings using modified procedures [73]. For fruit analysis, cuticle layers (1 cm^2^) were isolated from fruit exocarp as described [74]. The samples collected from each mutant line were considered as a biological replicate and four biological replicates were used. Samples were freeze-dried for 12 h, ground, and resuspended in 2 mL of 1 N methanolic HCl solution [75]. Depolymerization was performed at 80°C for 2 h, then reactions were cooled to room temperature and terminated with 2 mL saturated NaCl solution. Methylated fatty acids and other monomers were extracted thrice with 2 mL hexane. The combined hexane phases were concentrated to 200 μL under nitrogen gas at 60°C. Monomers were derivatized using 20 μL each of N,O-Bis(trimethylsilyl)trifluoroacetamide (BSTFA) and pyridine at 70°C for 40 min. Methyl heptadecanoate (C17:0) and ω-pentadecalactone (C15:0) served as internal standards. Cutin analysis was performed using an Agilent 7890B GC Network system equipped with a mass spectrometer, flame ionization detector, and DB-5MS column (30 m × 0.25 mm, 0.25 μm film; Agilent). Fruit samples were injected in split mode (5:1), while trichome samples were injected splitless. The temperature program was: 50°C for 2 min, ramped at 40°C/min to 200°C (2 min hold), then 3°C/min to 310°C (30 min hold). Cutin monomers were quantified by comparing integrated peak areas with internal standard peaks of known concentrations.

### Cuticle permeability assays

Leaf cuticle permeability was assessed by immersing detached leaves in 3 mL of 0.05% (w/v) TB solution for 30 min using a 5 mL syringe. Fruit cuticle permeability was evaluated following a former protocol [74], where MG and RR fruits were partially submerged (25% surface area) in 1% (w/v) TB solution for 4 h. After treatment, both leaves and fruits were gently rinsed with water to remove excess dye.

### Phylogenetic tree construction

*SlLACS1* and *SlLACS2* homologs were identified from 220 green plant genomes using HMM Model [76] and BLAST [77]. Amino acid sequences were aligned using MUSCLE [78] with default parameters. Phylogenetic analysis was performed using RAxML/8.0.6 [79] with maximum likelihood method and 1000 bootstrap replicates (parameters: -f a -x 12 345 p 12345 -# 1000 m PROTGAMMAAUTO –auto-prot = bic). The resulting phylogenetic tree was visualized using the chiplot online tool (https://www.chiplot.online/#).

### Pollen viability and morphology assay

Pollen viability was assessed using Alexander staining as described with minor modifications [80]. Anthers from at least three opened flowers per plant were suspended in ddH_2_O (200 μL per anther). The pollen suspension (5 μL) was mixed with 10% Alexander solution (15 μL; Solarbio Science and Technology, Beijing, China) on microscope slides and covered immediately. After 5 min staining, samples were examined using a Nikon ECLIPSE Ei bright-field microscope equipped with a Nikon digital camera. Purple-red stained pollen grains were counted as viable, while unstained grains were considered inviable. At least five microscopic fields were analyzed per biological replicate. For surface structure analysis, dried pollen from opened flowers was mounted on conductive double-sided tape, gold-coated, and examined using a Hitachi SU-8010 SEM.

### Statistical analysis

Statistical analyses were conducted using GraphPad Prism v10. Comparisons between wild type and mutants were performed using unpaired Student's *t*-tests.

### Accession numbers

Sequence data from this article are available in Solanaceae Genomics Network (https://solgenomics.net/), Phytozome 13 (https://phytozome-next.jgi.doe.gov/), PhycoCosm (https://phycocosm.jgi.doe.gov/) and Figshare (https://figshare.com/articles/dataset/Genomes_of_subaerial_Zygnematophyceae_provide_insights_into_land_plant_evolution/9911876/1). The tomato genes are under the following accession numbers: *SlLACS1* (Solyc01g079240); *SlLACS2* (Solyc01g109180); *CER1* (Solyc01g088400); *PECTIN2* (Solyc01g091050); *PIPK6* (Solyc01g096410); *CSY* (Solyc01g097930); *MYB94* (Solyc02g067340); *MYB* (Solyc02g076670); *GDSL* (Solyc02g077330); *OBL1* (Solyc02g090930); *KCS6* (Solyc02g063140); and *KCS11* (Solyc03g005320). The Algal genes are under the following accession numbers: *CreLACS* (Cre13.g566650.t1.2); *MvLACS* (Mv16422-RA.1); and *MeLACS* (ME000128S00185). The raw sequence data of RNA-seq has been deposited in the Sequence Read Archive in National Center for Biotechnology Information, under the accession number PRJNA1185636.

## Supplementary Material

Web_Material_uhaf114

## Data Availability

All data are incorporated into the article and its online supplementary material.
