# Supplementary material for: Evolutionary diversification of acyl-CoA synthetases underpins hydrophobic barrier formation across diverse tomato tissues and beyond
Source: Hortic Res. 2025 Apr 28;12(8):uhaf114. doi: 10.1093/hr/uhaf114 (PMC12247511; doi:10.1093/hr/uhaf114)
Supplement: Web_Material_uhaf114 [file web_material_uhaf114.zip › Supplemental Figures S1-17.pdf]

Supporting information

Evolutionary diversification of acyl-CoA synthetases underpins the hydrophobic barrier formation across diverse tomato tissues and beyond

Jianfeng Jin, Qiyu He, Xiangyi Feng, Jianjing Wang, Tao Lyu, Jinheng Pan, Jiarong Chen, Shan Feng, Xing-xing Shen, Jingquan Yu, Robert L Last, and Pengxiang Fan*

This file includes:

Figures S1 to S17

Other Supplementary Materials for this manuscript include the following:

Tables S1 to S7

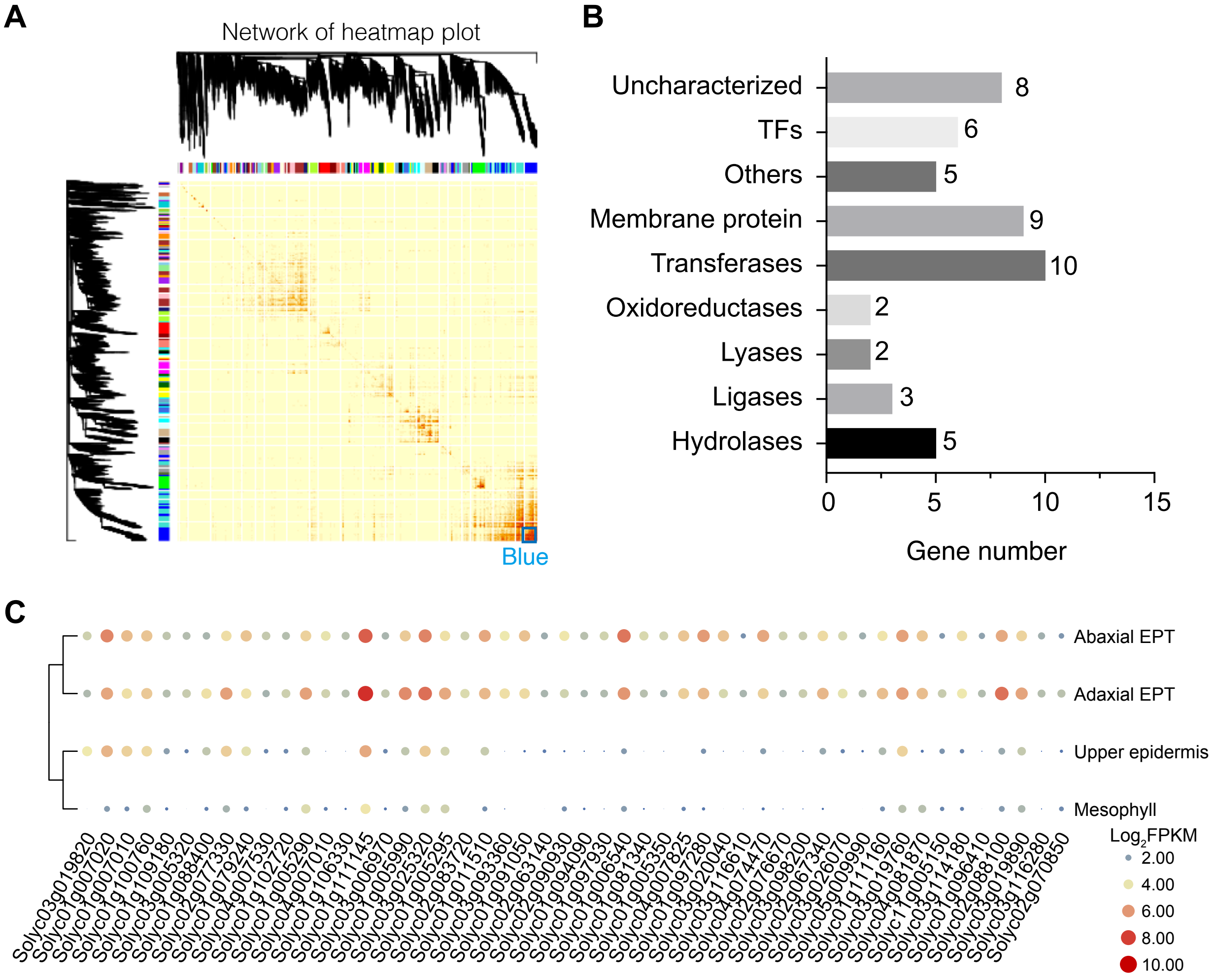

Figure S1. Identification of key genes in tomato leaf epidermis by weighted gene co-expression network analysis (WGCNA). A) Heatmap of the topological overlap matrix (TOM) displaying gene interconnectivity, with lighter to darker shades indicating increasing topological overlap. Dendrograms show gene clustering and initial module assignment. The blue box highlights a module of 1,061 highly correlated, adaxial EPT-enriched genes. B) Further refinement based on the criteria (FPKM ≥ 10 and log2[adaxial EPT/mesophyll] ≥ 2) narrowed the genes in the blue module to 50 genes that belong to multiple gene families. C) Expression profiles of fifty candidate genes visualized as a heatmap across different leaf tissue sections, including adaxial EPT, abaxial EPT, mesophyll, and upper epidermis.

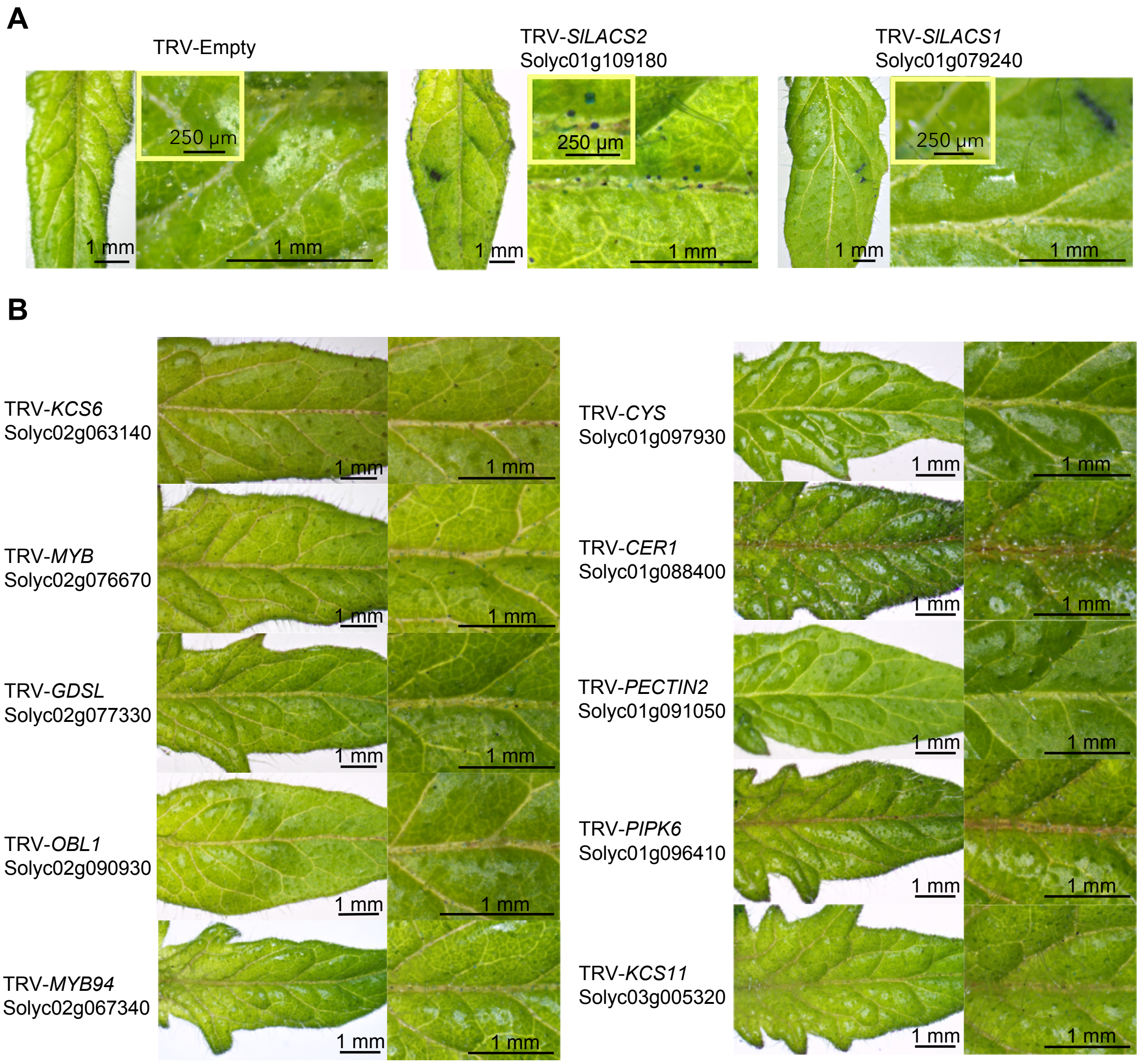

Figure S2. TB staining assay of leaves from 12 gene silencing plants generated by VIGS. A) The tomato leaves transiently silencing *SlLACS1* (Solyc01g079240) and *SlLACS2* (Solyc01g109180) by VIGS displayed distinct blue dots on their surface following TB staining, contrasting with the unstained M82 control. Scale bars = 1 mm. At least three tomato seedlings transiently silencing *SlLACS1* and *SlLACS2* were examined and showed similar phenotype after TB staining. The yellow rectangles represent enlarged images from leaves of TRV-empty control, TRV-*SlLACS2*, and TRV-*SlLACS1* plants after TB staining. Scale bars = 250 μm. B) Genes for VIGS including Solyc02g063140 (3-ketoacyl-CoA synthase 6, *KCS6*), Solyc02g076670 (*MYB*), Solyc02g077330 (*GDSL*), Solyc02g090930 (*OBL1*), Solyc02g067340 (*MYB94*), Solyc01g097930 (Cysteine synthase, *CYS*), Solyc01g088400 (Very-long-chain aldehyde decarbonylase, *CER1*), Solyc01g091050 (PECTINESTERASE2, *PECTIN2*), Solyc01g096410 (Phosphatidylinositol 4-phosphate 5-kinase 6, *PIPK6*), Solyc03g005320 (3-ketoacyl-CoA synthase 11, *KCS11*). At least three tomato seedlings transiently silencing genes were examined and showed no phenotype compare to control TRV-Empty after TB staining. Scale bars = 1 mm.

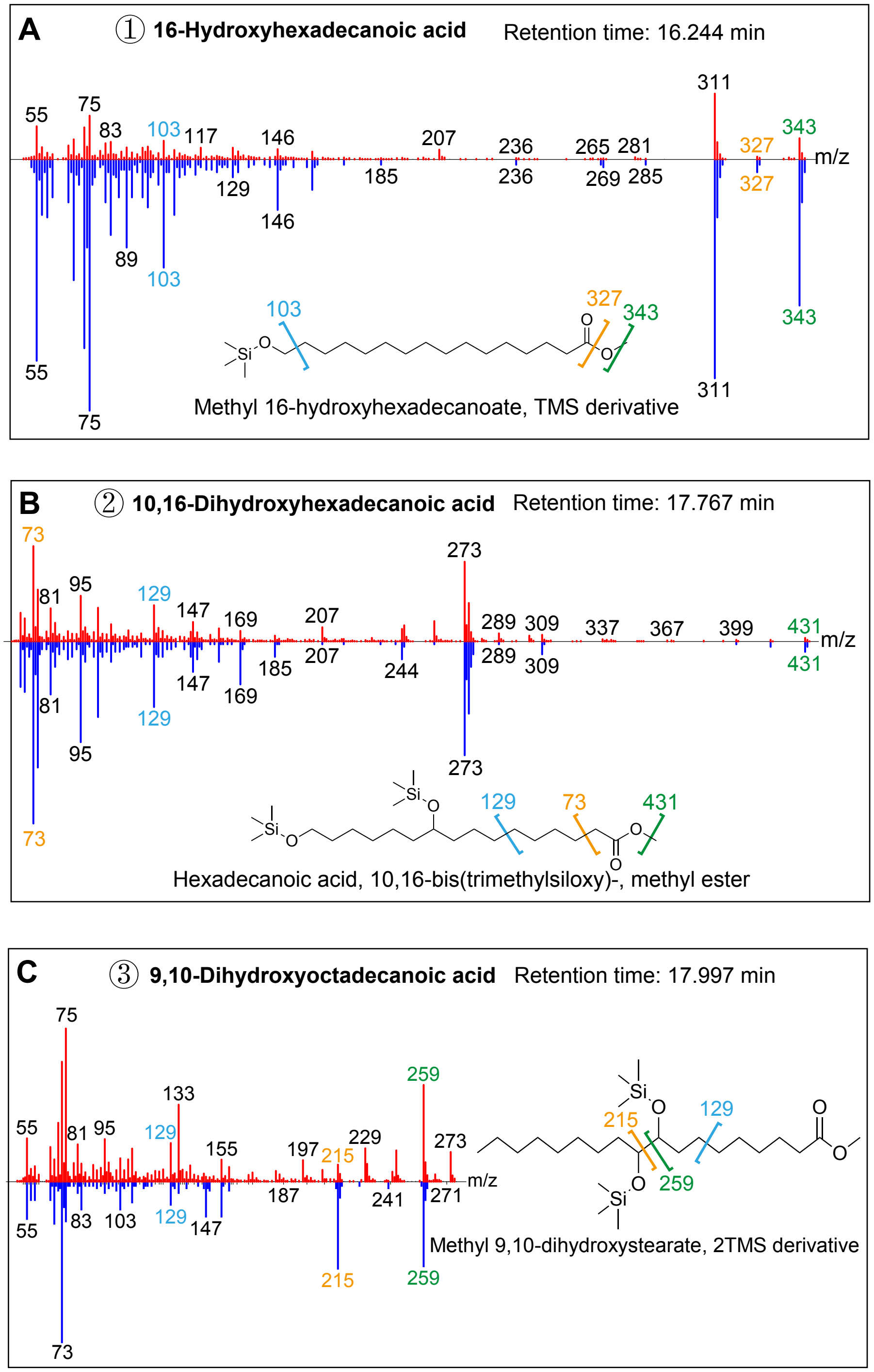

Figure S3. Analysis of cutin monomers structure by searching the mass spectra of NIST GC library. A-C) The proposed representative mass spectral fragmentation pattern for the trimethylsiloxy (TMS) derivatives of methyl esterification of 16-Hydroxyhexadecanoic acid (A), 10,16-Dihydroxyhexadecanoic acid (B), and 9,10-Dihydroxyoctadecanoic acid (C), respectively. Square brackets and color digitals represent the location of compound fragmentation and m/z of fragments.

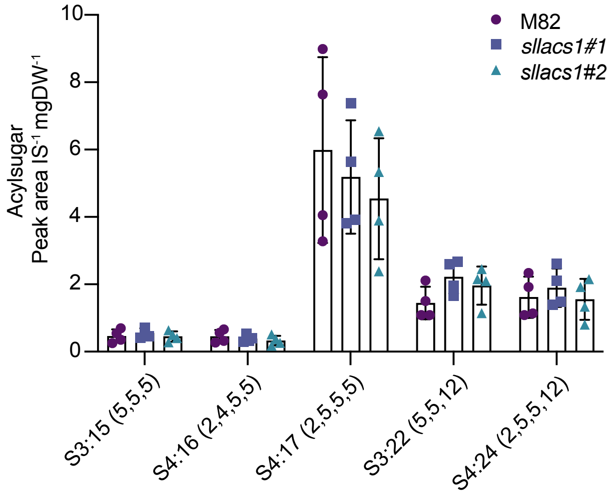

Figure S4. Analysis of major leaf acylsugar content in CRISPR/Cas9-generated *sllacs1* mutants compared to M82 control plants (Supports Figure 6). Acylsugar levels are shown as LC/MS peak areas normalized to internal standard (IS) values across four independent homozygous lines per mutant. Data are presented as means ± SD (n = 4); Unpaired *t*-test was performed to determine the statistical significance.

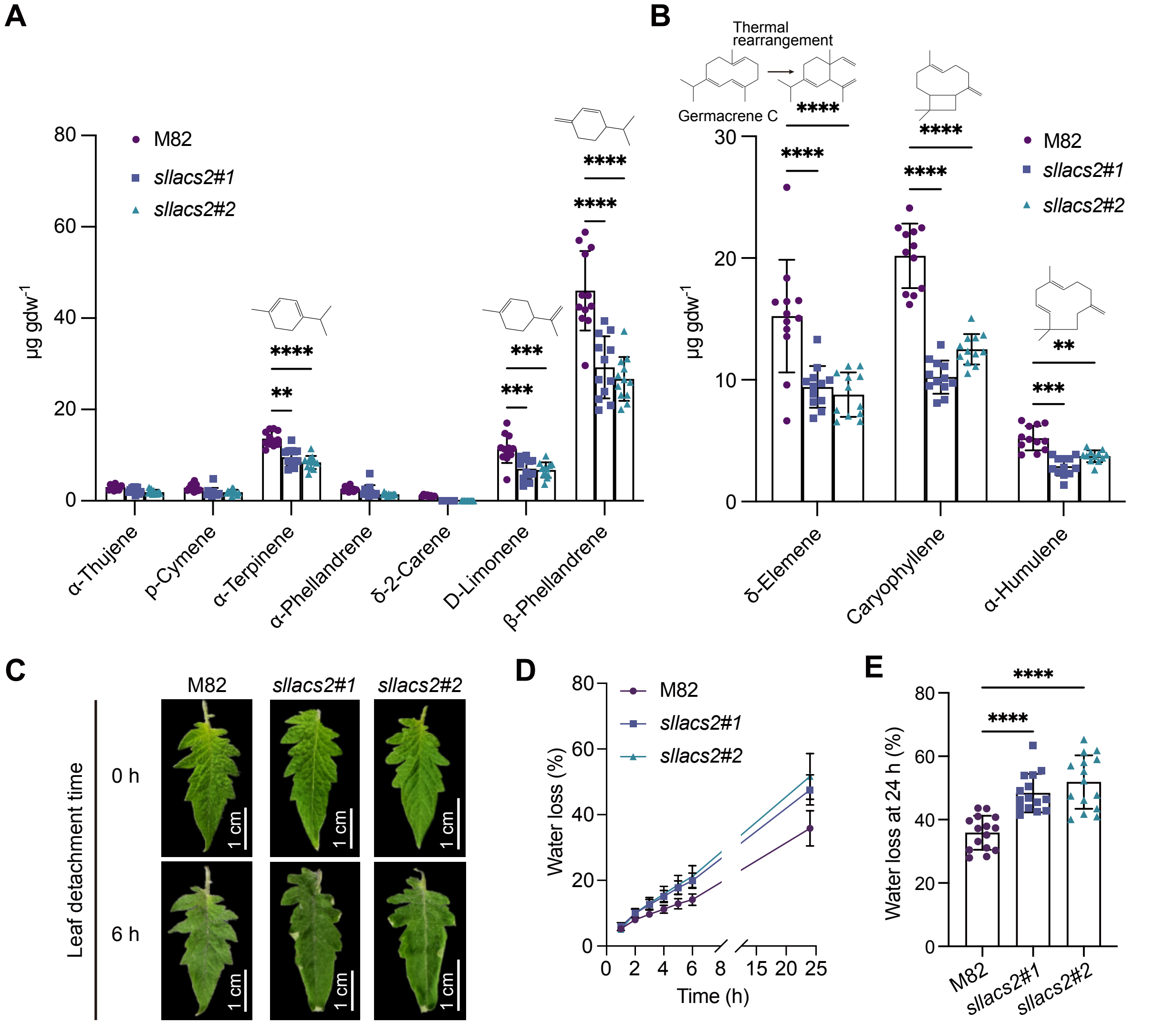

Figure S5. Analysis of terpenoid content and water loss rate of leaves from M82, *sllacs2#1*, and *sllacs2#2* plants. A-B) Analysis of monoterpenes (A) and sesquiterpene (B) content by GC-MS. Note that δ-elemene is not a direct product of trichomes but results from the thermal rearrangement of germacrene C within the GC column. Data are presented as means ± SD (n=12). C) Images depict the leaves of M82, *sllacs2#1*, and *sllacs2#2* tomato plants taken 6 hours post-detachment. D) The water loss rates of leaves from M82 and *sllacs2* mutants were recorded at various intervals following detachment at room temperature. E) Water loss in leaves from M82, *sllacs2#1*, and *sllacs2#2* was measured 24 hours after detachment. Data are shown as mean ± SD (n = 15). Statistical significance was determined using an unpaired *t*-test, with significance indicated by * $P < 0.05$, ** $P < 0.01$, *** $P < 0.001$, **** $P < 0.0001$.

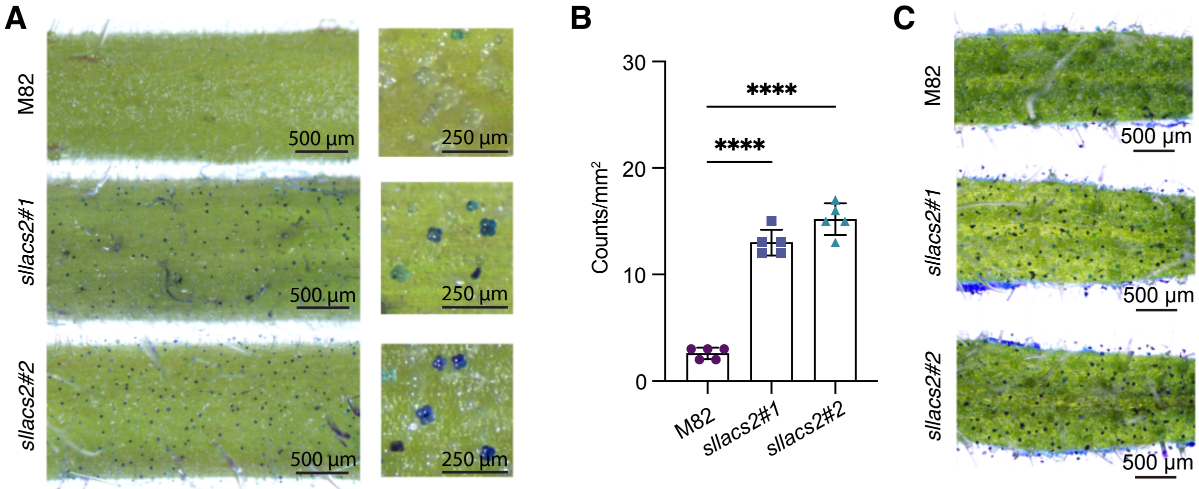

Figure S6. Permeability analysis of type VI trichomes from stem and sepal in M82, *sllacs2#1*, and *sllacs2#2* lines. A) TB staining of tomato stems presents similar patterns of tiny blue dots on the *sllacs2* mutant surfaces (left panel), with an enlarged view confirming TB-stained type VI trichomes (right panel). Scale bars = 500 µm (left panel) an 250 µm (right panel). B) Quantification of TB-stained type VI trichomes on the stems from M82, *sllacs2#1*, and *sllacs2#2* plants. C) TB staining on trichome-rich sepals shows consistent trichome staining in *sllacs2* mutants. Scale bars = 500 µm. Data are presented as means ± SD (n=5); Unpaired t-test was performed and the significant differences are represented by black asterisks: **** $P < 0.0001$.

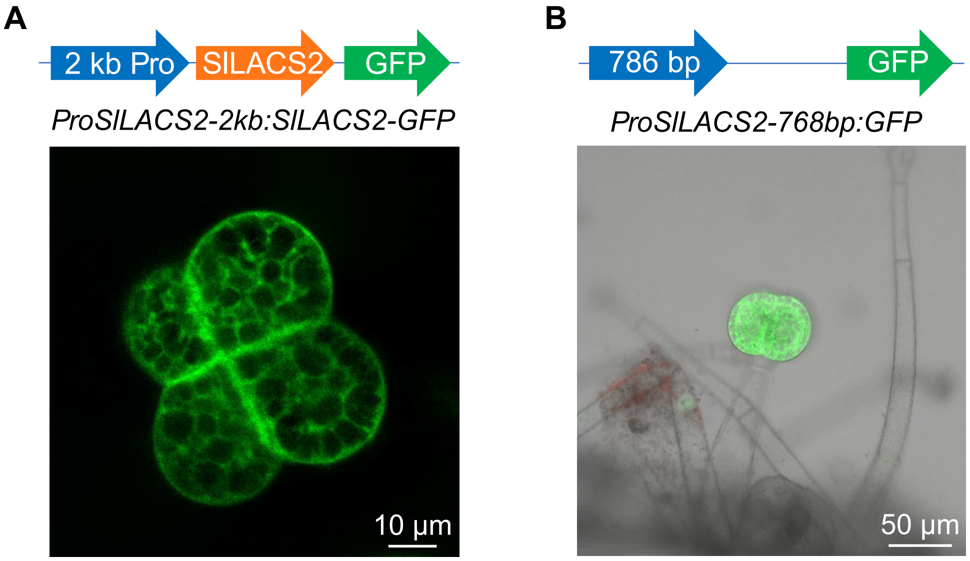

Figure S7. Further analysis *SlLACS2* tissue-specific localization. A-B) GFP fluorescence was detected in the glandular heads of type VI trichomes in tomato plants stably transformed with *ProSlLACS2-2kb:SlLACS2-GFP* A) and *ProLACS2-786bp:GFP-GUS* B) constructs. Blue arrows represent 2 kb A) and 768 bp B) *SlLACS2* promoter, respectively. Orange arrow represents the genomic sequence of *SlLACS2* including introns and exons. Green arrows represent GFP. Scale bars = 10 µm (A) and 50 µm (B).

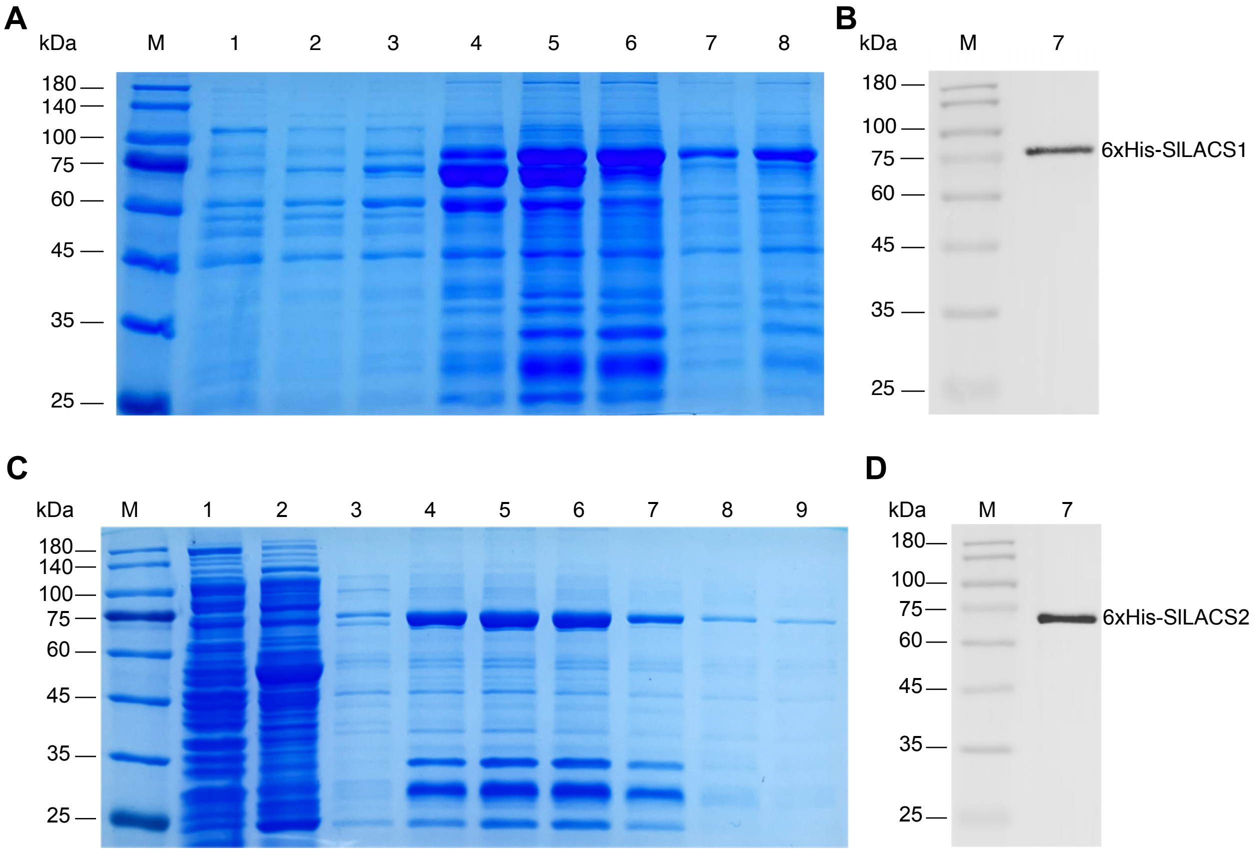

Figure S8. Expression and purification of recombinant SlLACS1 and SlLACS2 in *E. coli*. A) SDS-PAGE analysis of SlLACS1 protein elution fractions using sequential imidazole concentration gradients. Lanes represent: lane M, protein marker; lane 1, the flow-through; lane 2, elution with 30 mM imidazole; lane 3, elution with 50 mM imidazole; lane 4, elution with 100 mM imidazole; lane 5 to 8, sequential fractions eluted with 250 mM imidazole. B) Western blot verification of purified recombinant SlLACS1 (lane 7) with anti-His antibodies. C) SDS-PAGE analysis of SlLACS2 protein elution fractions using sequential imidazole concentration gradients. Lanes represent: lane M, protein ladders; lane 1, the flow-through; lane 2, elution with 30 mM imidazole; lane 3, elution with 50 mM imidazole; lane 4, elution with 100 mM imidazole; lane 5, elution with 150 mM imidazole; lane 6 to 9, sequential fractions eluted with 250 mM imidazole. D) Western blot verification of purified recombinant SlLACS2 (lane 7) with anti-His antibodies.

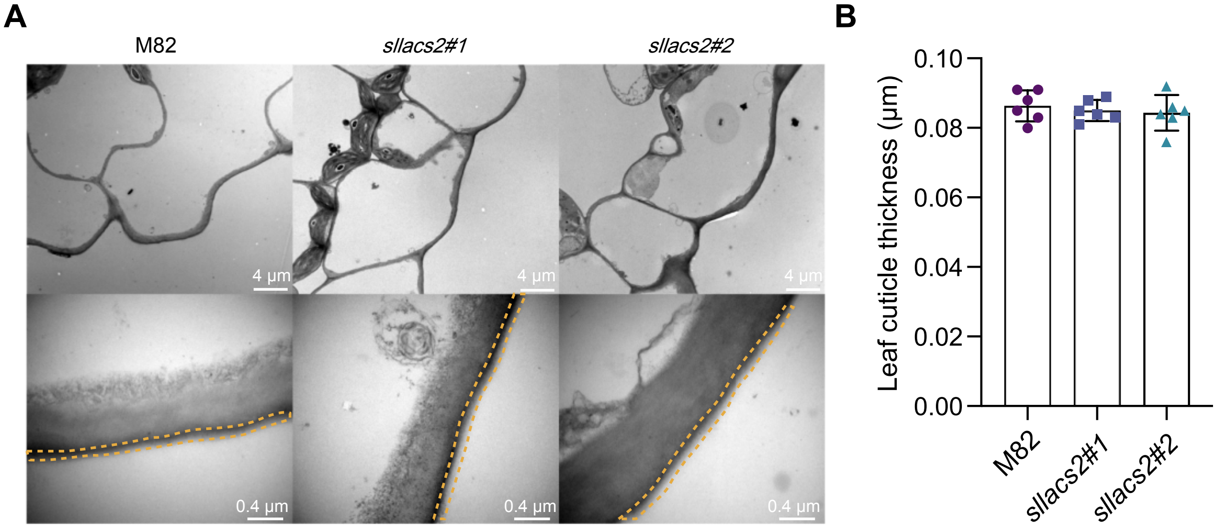

Figure S9. Quantification of leaf cuticle thickness in M82 and *sllacs2* mutants using transmission electron microscopy (TEM). A) TEM images showing the leaf cuticle layers (indicated by dotted yellow lines) of M82 wild-type and *sllacs2* mutants. B) Cuticle thickness measurements analyzed using ImageJ software. Values represent means ± SD (n=6); statistical significance was determined by unpaired *t*-test.

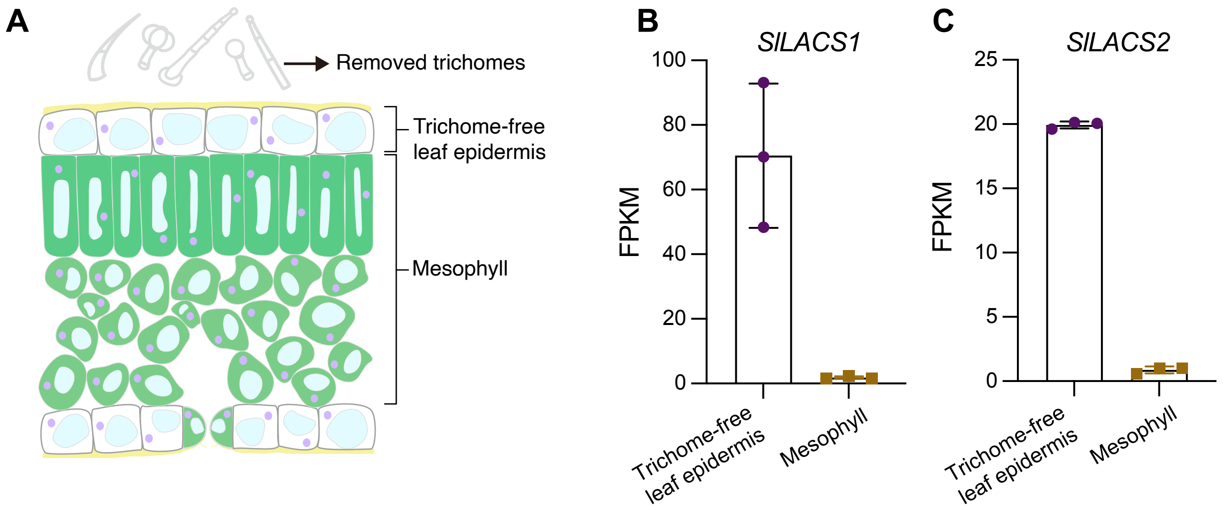

Figure S10. Expression profiles of *SlLACS1* and *SlLACS2* reveal enrichment in trichome-free leaf epidermis compared to mesophyll tissue. A) Schematic representation of trichome-free leaf epidermis tissue harvested for comparative transcriptome analysis. B) FPKM values for *SlLACS1* expression in trichome-free leaf epidermis versus mesophyll tissue. C) FPKM values for *SlLACS2* expression in trichome-free leaf epidermis versus mesophyll tissue.

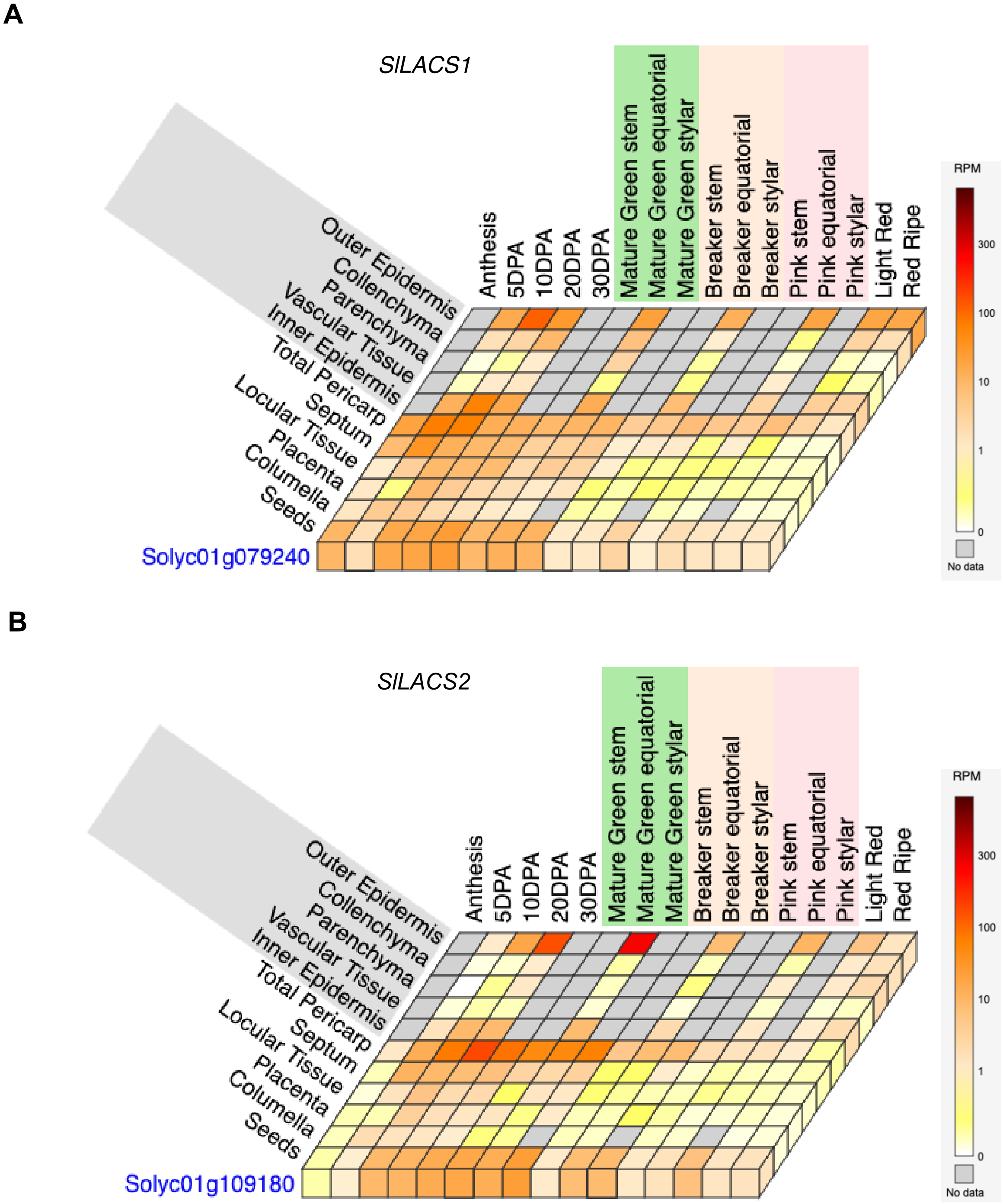

Figure S11. Tissue-specific expression patterns of *SlLACS1* (Solyc01g079240) and *SlLACS2* (Solyc01g109180) during tomato fruit development. A-B) Heatmaps showing the expression profiles of A) *SlLACS1* and B) *SlLACS2* across fruit tissues (outer epidermis, collenchyma, parenchyma, vascular tissue, inner epidermis, total pericarp, septum, locular tissue, placenta, columella, and seeds) at developmental stages from anthesis through fruit ripening (Anthesis, 5, 10, 20 DPA, Mature, Breaker, Pink, Light Red, and Red Ripe). DPA: days post-anthesis. Data retrieved from Tomato Expression Atlas (https://tea.solgenomics.net/).

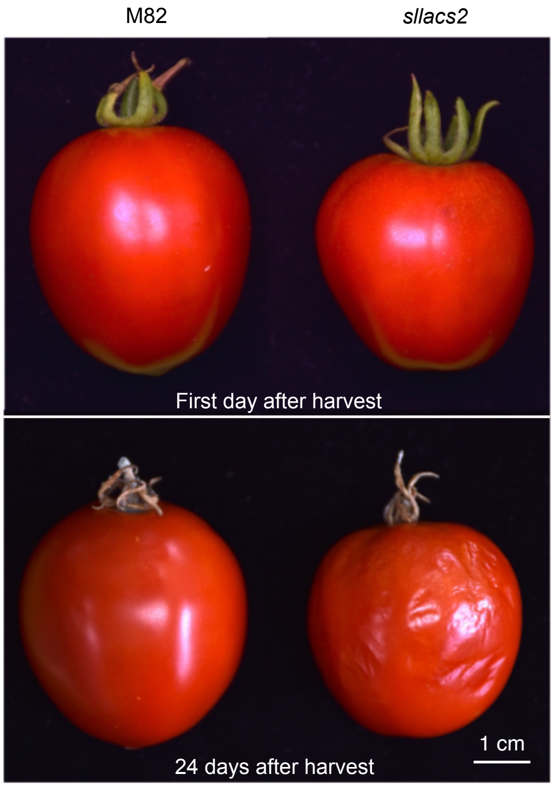

Figure S12. Photographic documentation of postharvest fruit dehydration in M82 and *sllacs2* tomatoes (Supports Figure 6). Red-ripe (RR) stage fruits were harvested with minimal handling to avoid peel damage and stored horizontally in plastic petri dishes under controlled climate conditions. Upper panel shows fruits at day 1 postharvest; lower panel shows fruits after 24 days of storage, demonstrating differential dehydration phenotypes. Scale bar = 1 cm.

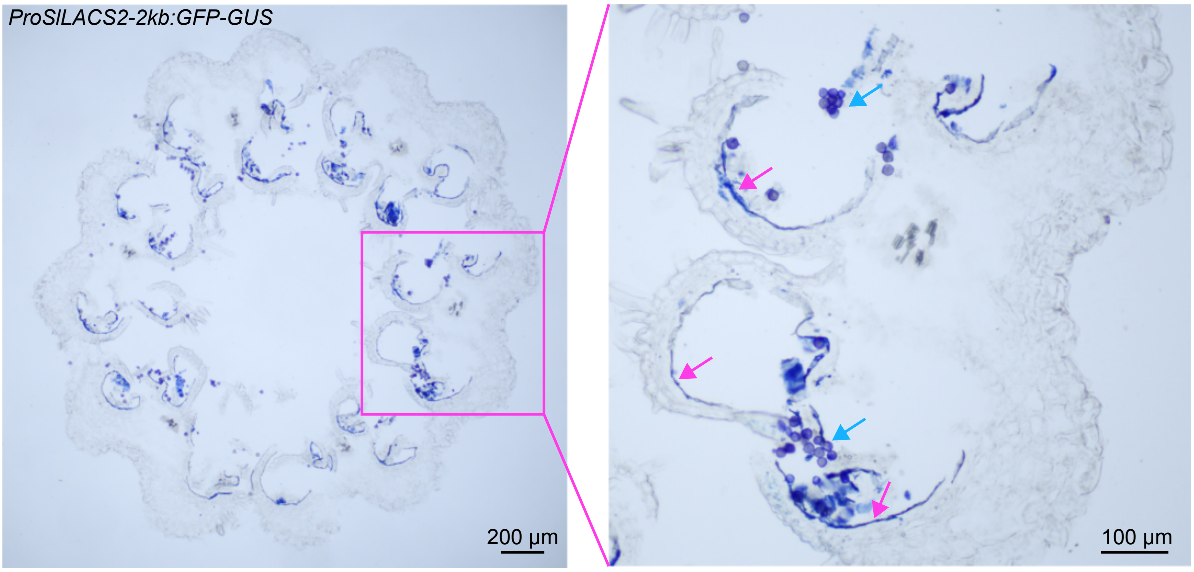

Figure S13. Visualization of *SlLACS2* expression in tomato anther. GUS activity in anther cross-sections from *ProSlLACS2-2kb:GFP-GUS* transgenic plants. Inset (rectangle) shows magnified view of anther section with GUS-stained tapetum (indicated by pink arrows) and pollen grains (indicated by blue arrows). Scale bars = 200 µm (left) and 100 µm (right).

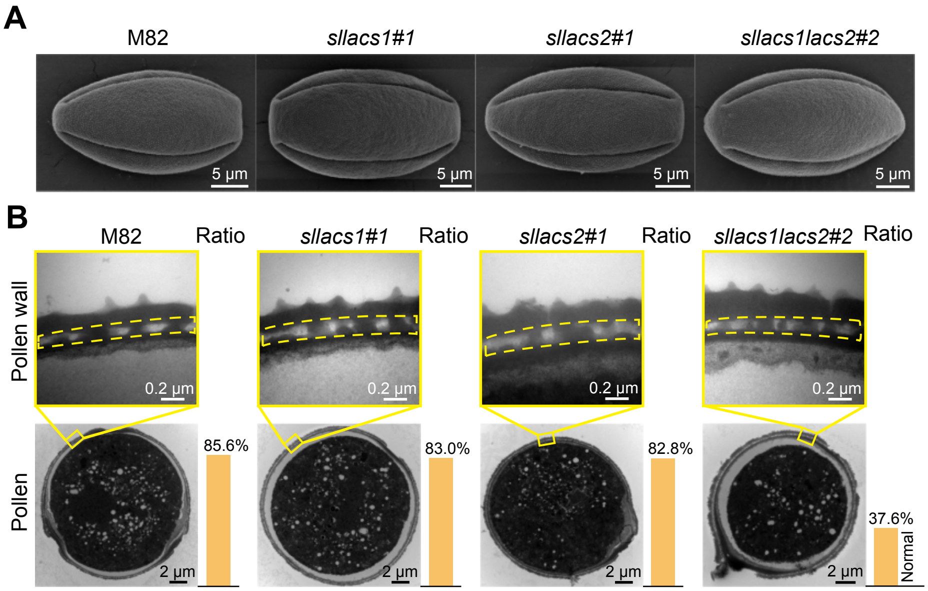

Figure S14. Analysis of normal pollen morphology in M82, single mutants, and double mutant plants. A) Higher magnification SEM images of normally developed pollen from all genotypes showed similar appearence. Scale bars = 5 µm. B) TEM cross-sections of normal pollen grains (bottom row) reveal structural differences, with particular attention to the pollen coat. Scale bars = 2 µm. Enlarged images of the pollen wall (top row) illustrate the pollen coat structure (delineated by the dotted yellow lines), which appears no structural difference in M82, single mutants, and double mutant plants. Scale bars = 0.2 µm. Orange bars represent the ratio of normal pollens from flowers of M82, *sllacs1#1*, *sllacs2#1*, and *lacs1lacs2#2* plants.

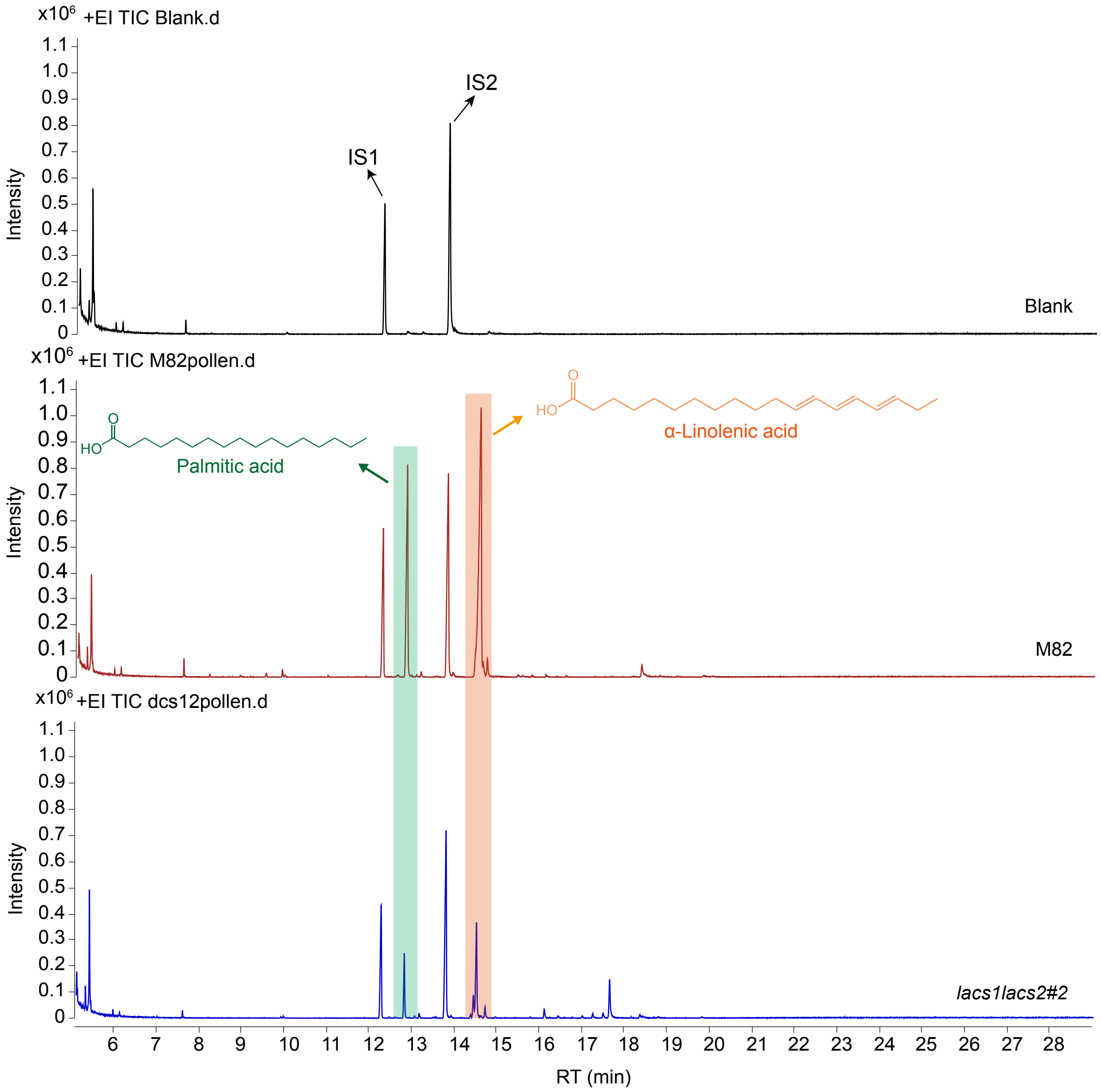

Figure S15. GC/MS analysis of pollen fatty acid composition in *lacs1lacs2#2* double mutant compared to M82 control. Fatty acids were extracted from equal numbers of opened flower pollens. Chromatograms show: top row - the blank sample with internal standards (IS1: ω-pentadecalactone, IS2: Methyl heptadecanoate, marked by black arrows); middle row - M82 fatty acid profile; bottom row - *lacs1lacs2#2* fatty acid profile. Peaks corresponding to palmitic acid (green shade) and α-linolenic acid (orange shade) are highlighted and annotated with their respective chemical structures.

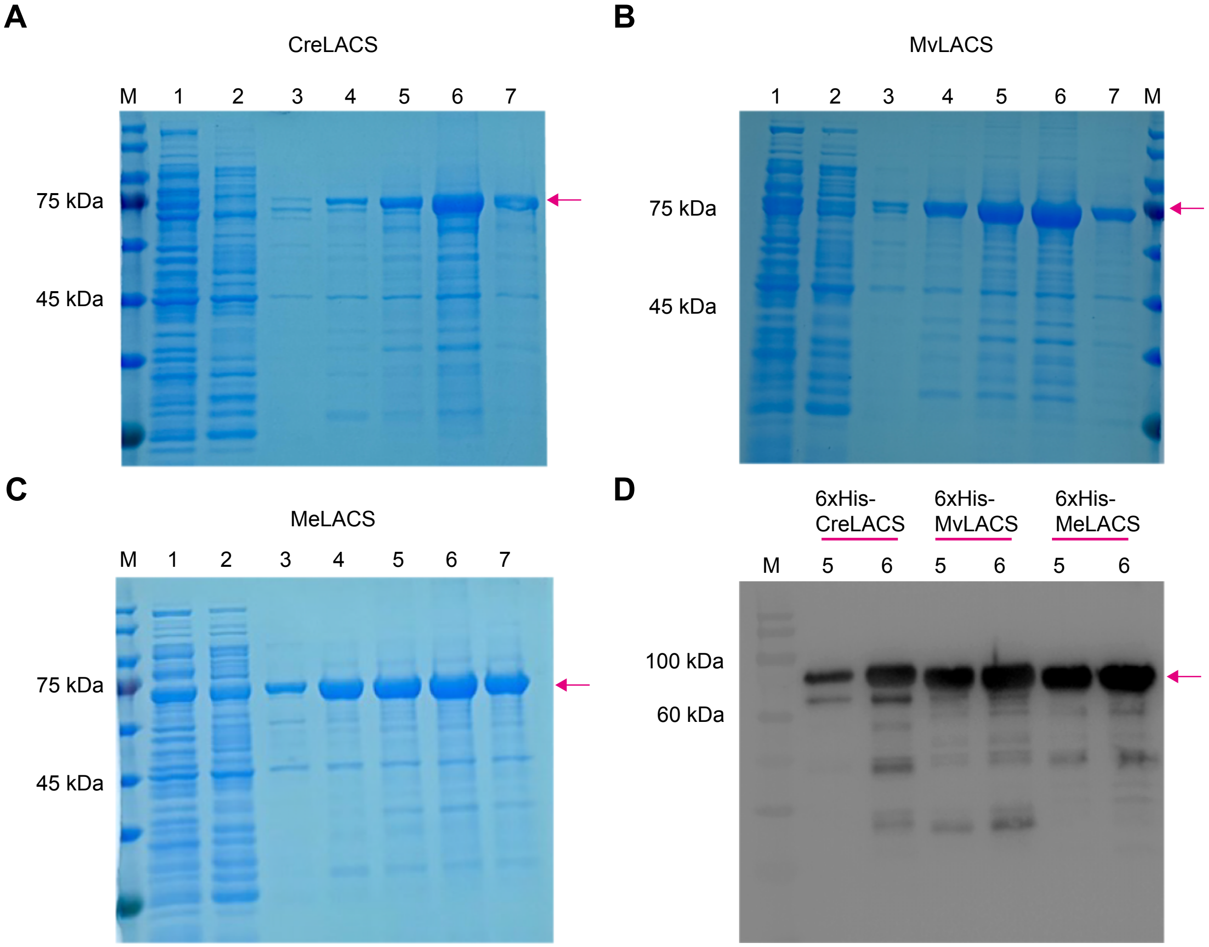

Figure S16. Expression and purification of recombinant LACS proteins from three algal species. A-C) SDS-PAGE analysis of protein elution fractions from A) *Chlamydomonas reinhardtii* (CreLACS), B) *Mesostigma viride* (MvLACS), and C) *Mesotaenium endlicherianum* (MeLACS) using increasing imidazole concentrations. The samples in each lane were as follows: lane M, protein marker; lane 1, the flow-through; lane 2, elution with 30 mM imidazole; lane 3, elution with 50 mM imidazole; lane 4 to 7, sequential fractions eluted with 250 mM imidazole. D) Western blot confirmation of purified recombinant proteins using anti-His antibodies. Samples from elution fractions 5 and 6 of CreLACS, MvLACS, and MeLACS were analyzed. Target proteins indicated by red arrows.

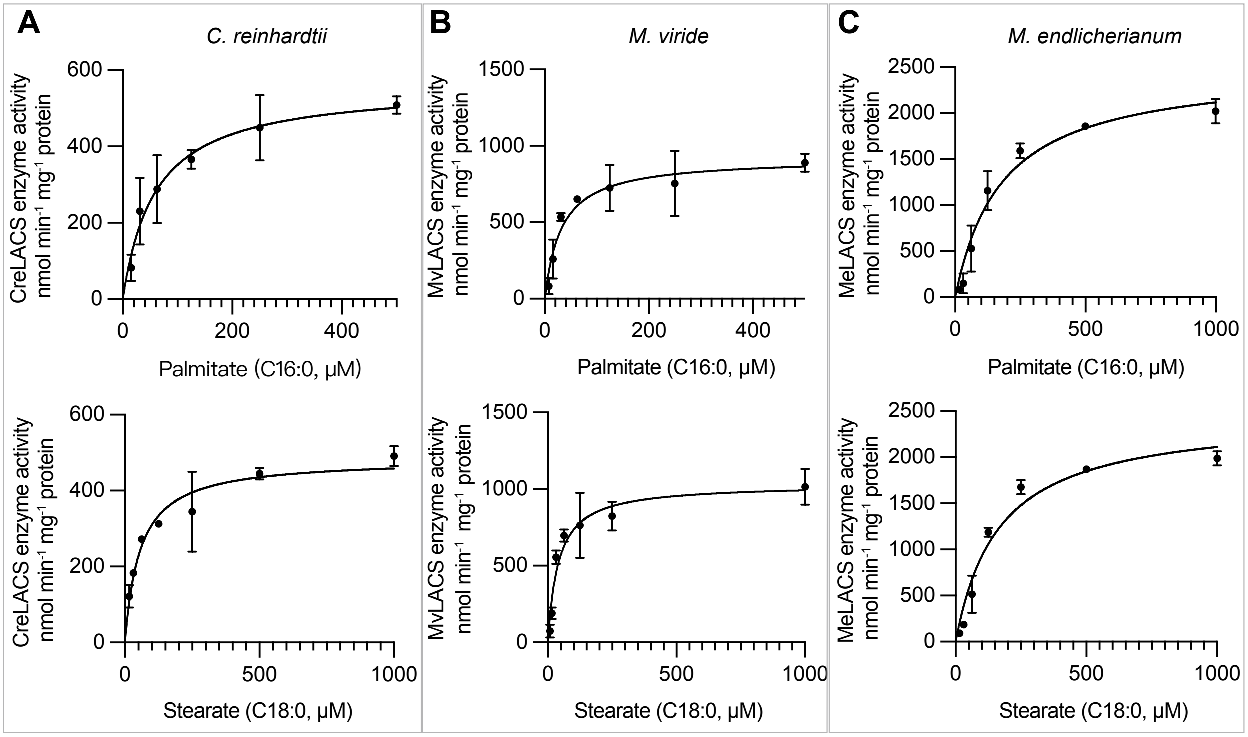

Figure S17. Michaelis-Menten kinetic analysis of recombinant algal LACS1/2 proteins. A-C) Enzyme kinetics plots for Chlamydomonas reinhardtii (CreLACS) A), Mesostigma viride (MvLACS) B), and Mesotaenium endlicherianum (MeLACS) C) using two fatty acid substrates: palmitate (C16:0, upper panels) and stearate (C18:0, lower panels).
